# Multiplex Protein Imaging through PACIFIC: Photoactive Immunofluorescence with Iterative Cleavage

**DOI:** 10.1021/acsbiomedchemau.3c00018

**Published:** 2023-04-28

**Authors:** Fei Ji, Moises Hur, Sungwon Hur, Siwen Wang, Priyanka Sarkar, Shiqun Shao, Desiree Aispuro, Xu Cong, Yanhao Hu, Zhonghan Li, Min Xue

**Affiliations:** † Department of Chemistry, University of California, Riverside, Riverside, California 92521, United States; ‡ Environmental Toxicology Graduate Program, University of California, Riverside, Riverside, California 92521, United States; § College of Chemical and Biological Engineering, Zhejiang University, Hangzhou, Zhejiang 310027, P.R. China; ∥ Martin Luther King Jr High School, Riverside, California 92508, United States; ⊥ Diamond Bar High School, Diamond Bar, California 91765, United States

**Keywords:** immunofluorescence, protein imaging, photocleavable linker, single-cell analysis, live-cell tracking

## Abstract

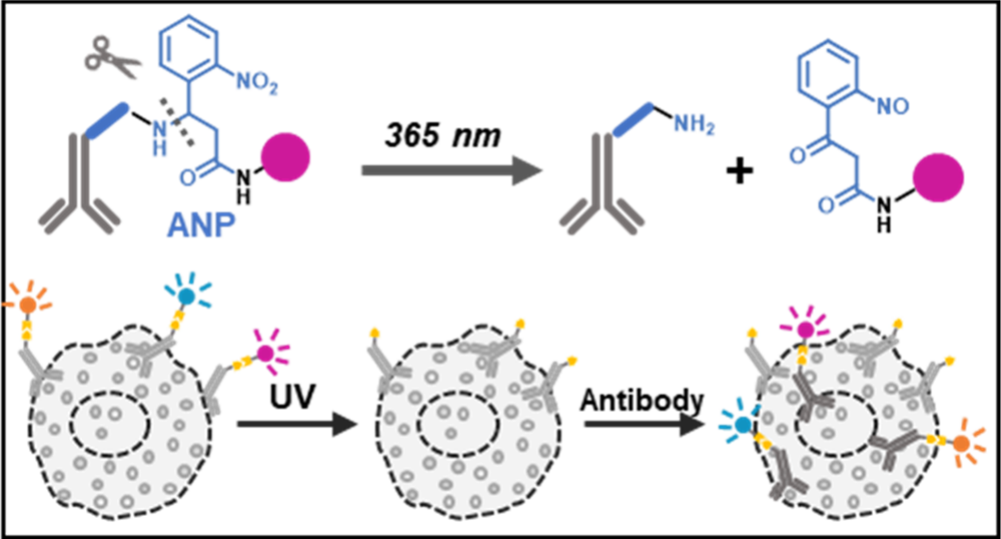

Multiplex protein imaging technologies enable deep phenotyping and provide rich spatial information about biological samples. Existing methods have shown great success but also harbored trade-offs between various pros and cons, underscoring the persisting necessity to expand the imaging toolkits. Here we present PACIFIC: photoactive immunofluorescence with iterative cleavage, a new modality of multiplex protein imaging methods. PACIFIC achieves iterative multiplexing by implementing photocleavable fluorophores for antibody labeling with one-step spin-column purification. PACIFIC requires no specialized instrument, no DNA encoding, or chemical treatments. We demonstrate that PACIFIC can resolve cellular heterogeneity in both formalin-fixed paraffin-embedded (FFPE) samples and fixed cells. To further highlight how PACIFIC assists discovery, we integrate PACIFIC with live-cell tracking and identify phosphor-p70S6K as a critical driver that governs U87 cell mobility. Considering the cost, flexibility, and compatibility, we foresee that PACIFIC can confer deep phenotyping capabilities to anyone with access to traditional immunofluorescence platforms.

## Introduction

Analysis of protein expression levels provides rich information about biological samples and is an indispensable part of biomedical research.^1−3^ An ever-expanding collection of studies has highlighted the value, and sometimes the need, of performing multiplex protein analysis, where detailed phenotyping promises a better understanding of the biological sample.^4−8^ The toolkit for achieving this goal contains a diverse selection, many of which are based on mass spectrometry^1,8,9^ and flow cytometry^10,11^ platforms. Despite their pivotal role, these platforms harbor some limitations. Mass spectrometry requires sophisticated sample preparation and data analysis that calls for strong expertise, and most researchers have limited access to high-multiplexity flow cytometers. More importantly, the spatial context of the original sample is often lost during the analysis. Although strategies such as microdissection can alleviate this problem,^12−14^ it is not a complete remedy.

On the other hand, traditional imaging-based methods, such as immunofluorescence (IF), can preserve spatial information with high fidelity. These methods are compatible with tissue samples, which is a crucial feature that enables wide applications in the clinic, assisting diagnosis and treatment. But, conventional IF workflows are limited by fluorophore spectral overlap,^15^ providing information on only a handful of biomarkers. Although linear unmixing algorithms can separate overlapping spectra,^16,17^ these methods require environment-insensitive fluorophores that do not change their spectra upon solvent polarity change, which can become a challenge in complex tissues samples.

To this end, a collection of iterative immunofluorescence tools emerged over the past decade, propelling fast advances in the field of multiplex spatial proteomics. Nevertheless, all these methods have their pros and cons, highlighting multilateral trade-offs between resolution, multiplexity, throughput, robustness, complexity, cost, and instrument access. For instance, photobleaching can erase signals on-demand and enable iterative imaging cycles.^18^ However, since various fluorophores and sample areas need to be bleached individually, this method is limited by the sample throughput. This limitation can be addressed by implementing whole-sample chemical bleaching^19,20^ and antibody stripping.^21^ Albeit effective, these processes often require harsh chemical treatments and can lead to epitope damage and sample degradation, affecting downstream signal fidelity. To avoid damaging sample integrity during signal removal, various milder methods have emerged, with representative examples including SAFE,^22^ CODEX,^23^ DNA displacement,^24^ and azide-based cleavable linkers.^25^ Meanwhile, signal amplification methods for higher sensitivity have also been developed, such as ImmunoSABER,^26^ CosMx,^27^ and cleavable fluorescent tyramide.^28,29^ Notably, some of these technologies have already shown commercial success, but the requirements for specialized reagents and instruments could become a barrier. In addition, many methods require DNA-antibody conjugates, which could cause spatial crowding, posing challenges for imaging protein complexes or multiple posttranslational modifications on the same protein. Moreover, mounting evidence has supported the necessity of integrating multiplex protein analysis with other bioanalytical tools,^30−33^ and the ability to connect the static phenotypical features with dynamic functional characteristics is particularly intriguing.^22,34−36^ Such exercises promise a more comprehensive understanding of the sample but, at the same time, introduce additional compatibility constraints. Therefore, expanding the arsenal of multiplex imaging tools remains necessary to support biomedical research better.

**Figure 1:**
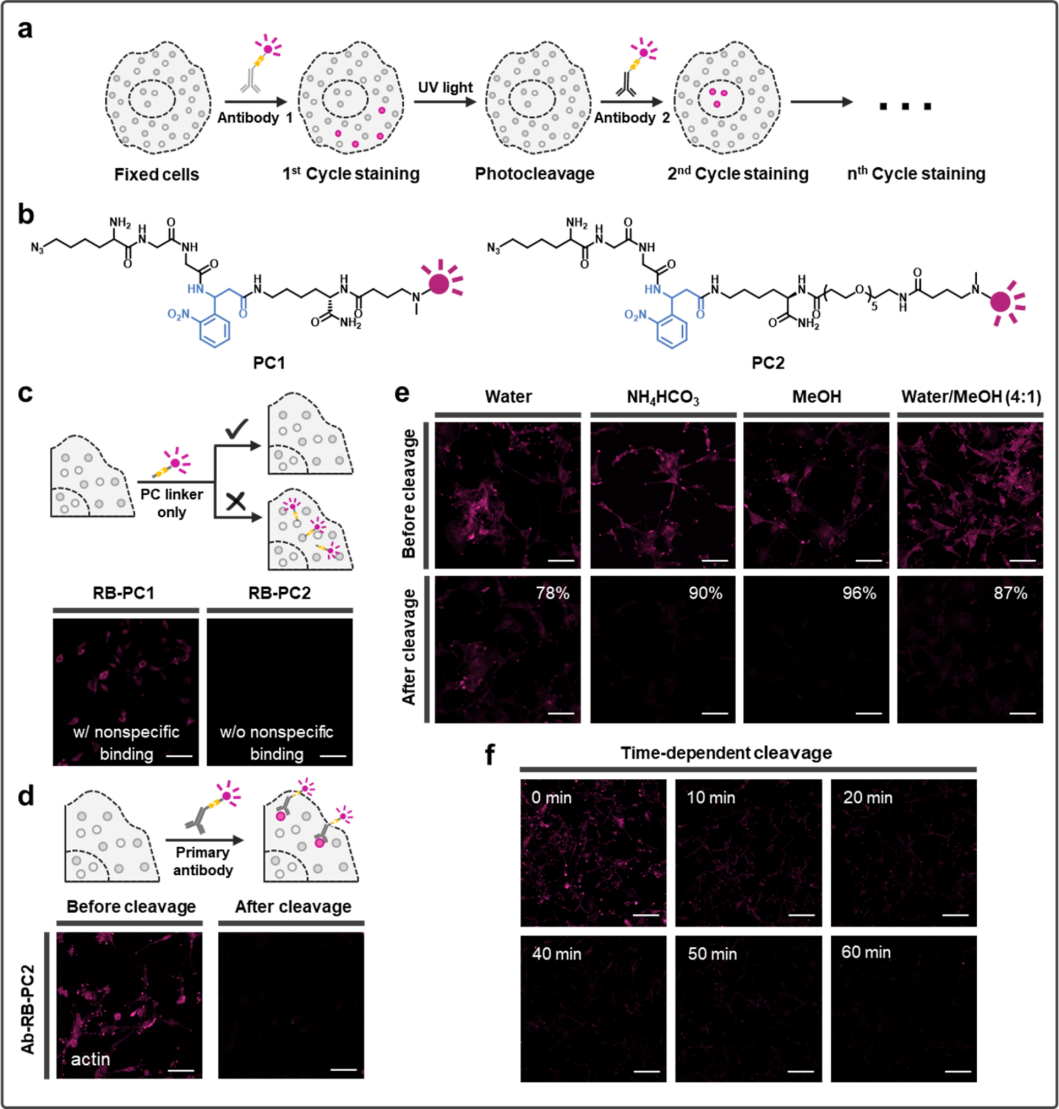
Design and optimization of PACIFIC. (a) Proteins in the cells can be quantified through fluorescent antibodies labeled with photocleavable (PC) linkers. After imaging, the PC linker and the fluorophore can be cleaved with UV light. This process allows iterative label-erase-label cycles of immunofluorescence. (b) Two PC linkers are designed. The azide tag allows antibody conjugation via a strain-promoted azide–alkyne cycloaddition (SPAAC) reaction, the ANP module works as the photoactive moiety, and a PEG5 segment is used in PC2 to increase the hydrophilicity. (c) Confocal images showing that RB-PC1 had a nonspecific binding with fixed cells and RB-PC2 could be easily removed. (d) Confocal images showing that the Ab-RB-PC2 was able to label the actin from U87 cells, and the signal can be erased by UV irradiation. (e) Cleavage tests of Ab-RB-PC2 in different media. (f) Time-dependent cleavage of Ab-RB-PC2 in argon-purged methanol. Scale bar, 50 μm.

Herein, we report a highly multiplex protein imaging technology, PACIFIC (photoactive immunofluorescence with iterative cleavage), whose multiplex capacity is conferred by two processes. First, antibodies are labeled with a panel of fluorophores via a linker, enabling simultaneous analysis of multiple proteins. Second, this linker is photocleavable, allowing iterative label–erase–label cycles that further boost the assay capacity (Figure 1a). We demonstrate that PACIFIC is compatible with fixed cells and FFPE tissue sections. We also show that PACIFIC can be combined with live-cell tracking and help unveil exciting biology.

## Results

### Developing a Photoactive Linker for Antibody Conjugation

We chose amino-3-(2-nitrophenyl)propionic acid (ANP) as the photoactive moiety. ANP is responsive to UV irradiation at 365 nm, with a high cleavage efficiency.^37,38^ With the goal of incorporating ANP into the linker between antibody and fluorophore, we considered four design criteria. First, the linker must be of sufficient length to separate the fluorophore from the antibody, which is necessary to prevent fluorescence quenching. Second, the cleaved linker/fluorophore moiety must exhibit minimum nonspecific binding and can be easily washed away. Third, the linker must be accessible via robust and simple synthetic procedures. Lastly, the conjugation strategy should require minimal purification. With those criteria, we designed a linker, PC1 (Figures 1b), that allowed antibody conjugation via a strain-promoted azide–alkyne cycloaddition (SPAAC) reaction.^39^

For proof of concept, we chose rhodamine B (RB) as a model fluorophore because it was cheap, stable, and bright. We synthesized RB-PC1 and confirmed its formation by MALDI mass spectroscopy (Figures S1, S2). We tested RB-PC1 on fixed U87 cells and found that RB-PC1 exhibited a substantial level of nonspecific binding (Figures 1c, S5), rendering it unsuitable for our design. To solve this problem, we installed a PEG5 segment and prepared the RB-PC2 linker (Figures 1b, S3, S4). As expected, RB-PC2 could easily be removed from fixed U87 cells (Figures 1c, S5).

We conjugated RB-PC2 to monoclonal rabbit-antihuman actin antibody, following established SPAAC conjugation protocols. This exercise was proven successful, as demonstrated by the immunofluorescence signal (Figure 1d). We then attempted to cleave the ANP linker by UV irradiation and remove RB fluorescence from the sample. As shown in Figure 1d, PC2 showed excellent photocleavage efficiency. As expected, antibodies labeled with RB-PC1 failed to respond under these conditions, which is consistent with its strong nonspecific binding (Figure S6). This result underscored the expected ability of PEG to minimize nonspecific interactions and supported our further studies using PC2.

### Optimizing the Conjugation and Photocleavage Conditions

Our SPAAC strategy requires modifying the antibody with highly hydrophobic DBCO groups. Consequently, there is a trade-off between the degree of labeling (DOL) and the propensity of antibody aggregation and denaturing. Therefore, we sought to identify the optimal DOL that maximizes the fluorescence brightness without affecting the antibody function. To attenuate the overall hydrophobicity, we employed a DBCO-PEG5-NHS labeling strategy, where the PEG5 group was expected to prevent aggregation. With this construct, we achieved a maximum DOL of ∼3.7 (Figure S7). Further increasing the DBCO amount caused severe precipitation and lowered antibody recovery yield. A similar trend was also observed in the corresponding fluorescence intensities (Figure S7).

Because the solvent environment often dictates photoactivity, we tested the cleavage of the ANP linker in different media to identify the best conditions (Figure 1e). We found that the RB-PC2 linker was cleaved at a 78% efficiency after 1 h of UV irradiation in water, and the addition of NH_4_HCO_3_ as a scavenger improved the cleavage efficiency to 90% (Figure S8). The best cleavage efficiency (96%) was achieved in methanol, which was implemented in subsequent studies. The cleavage efficiency in methanol was also time-dependent, where 60% of cleavage occurred within 10 min of UV irradiation (Figures 1f, S9). It is also worth noting that purging the solvent with argon helped boost the efficiency (Figure S10).

### Implementing PACIFIC on Fixed Cells

**Figure 2:**
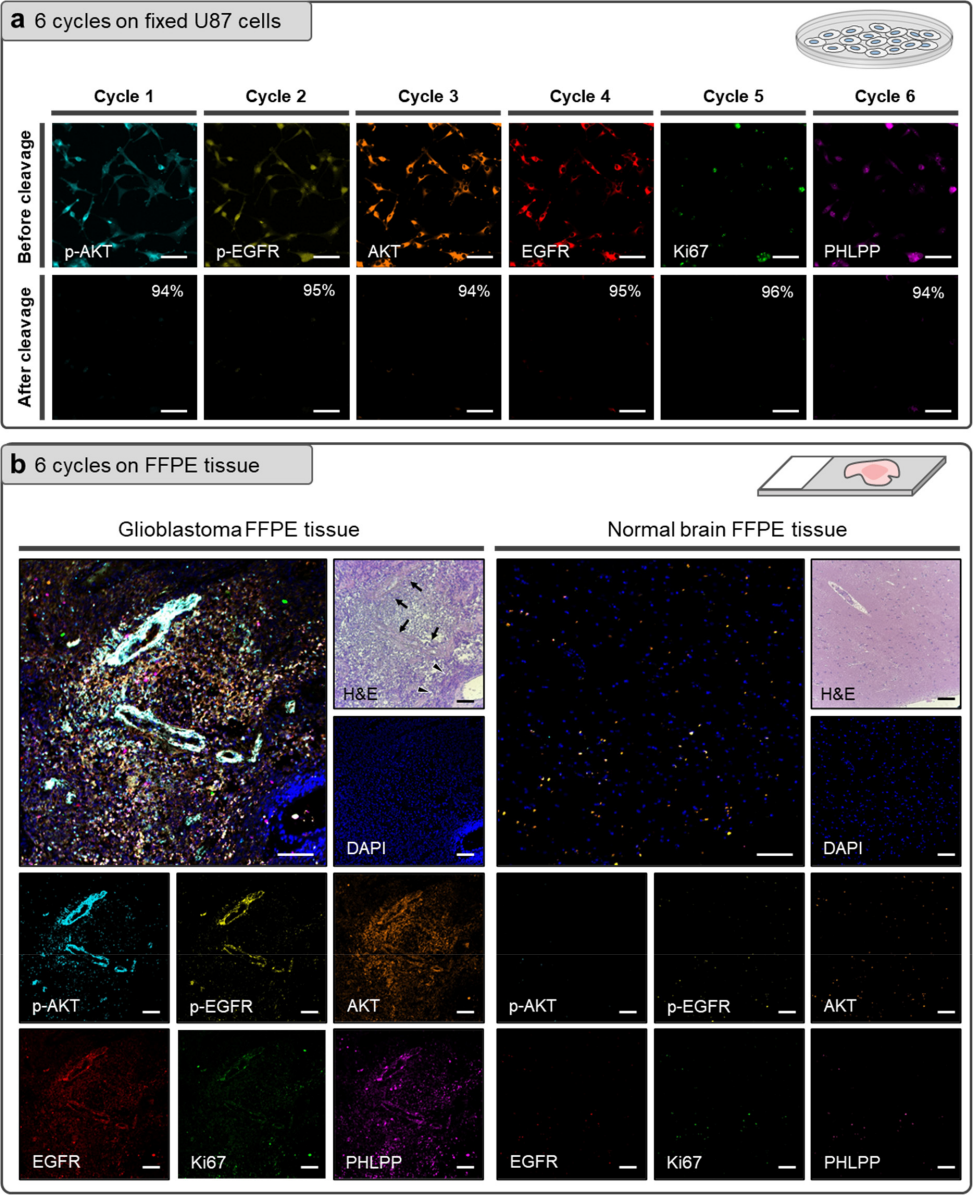
Implementing PACIFIC on fixed cells and FFPE tissue samples. (a) Confocal images showing six cycles of immunofluorescence on fixed U87 cells, both before and after cleavage. Scale bar, 50 μm. (b) Confocal images showing six cycles of immunofluorescence on glioblastoma and normal brain FFPE tissue samples, which demonstrated a stark difference in protein expression. In the H&E staining image of tumor sample, arrows mark the regions with significant vascular endothelial proliferation where high levels of phosphoproteins, as well as the proliferation marker Ki67, were observed, and triangles mark the pseudopalisading region that exhibited very low levels of signals. Scale bar, 100 μm.

With the optimized labeling and cleavage conditions, we implemented PACIFIC in fixed U87 cells. We conjugated six types of antibodies to the RB-PC2 linker and collected immunofluorescence images following the label-erase-label cycle described above. As shown in Figure 2a, all six antibodies successfully produced immunofluorescence, and the fluorescence signals at each iteration were effectively removed by photocleavage. The photocleavage efficiency remained consistent over the six cycles (Figure S11). Notably, the immunofluorescence signals were consistent with the expected distribution of the target proteins. For instance, the Ki67 signal was found only in the nucleus. More interestingly, we observed that most AKT proteins were phosphorylated, echoing the hyperactive PI3K-AKT pathway in U87 cells.^40^ Cytosolic EGFR proteins were mostly nonphosphorylated, whereas nuclear ones were phosphorylated, consistent with p-EGFR’s role in assisting transcription.^41^ These results highlight PACIFIC’s ability to obtain both expression and phosphorylation levels of the same protein in the same cells and provide subcellular spatial information.

### Implementing PACIFIC on FFPE Tissue Samples

To further demonstrate the application of PACIFIC, we tested its performance on FFPE tissue samples. Here, we analyzed tumor and normal brain tissue samples collected from the temporal lobe of the same glioblastoma patient. As shown in Figure 2b, we were able to obtain high-quality images from these tissue samples using PACIFIC. We observed a stark difference in overall signal intensities between the tumor and normal tissues, consistent with the proliferation status of those cells. In the tumor tissue, levels of phosphoproteins correlated well with the proliferation marker Ki67, with the strongest signals appearing in regions with significant vascular endothelial proliferation. On the other hand, the pseudopalisading region exhibited very low levels of signals, which is consistent with its necrotic/apoptotic nature.

PACIFIC also revealed interesting phenotypical heterogeneity in the sample. Most of the PHLPP+ cells exhibited low p-AKT, which is consistent with PHLPP’s function of dephosphorylating AKT.^42^ However, some cells showed strong signals of both p-AKT and PHLPP (Figure S12), indicating unsuccessful suppression of p-AKT by PHLPP in these cells. Similar unexpected colocalizations also existed between Ki67 and PHLPP in these cells. In addition, we identified cells with weak p-AKT and p-EGFR signals but strong Ki67 signals, suggesting that some other signaling pathways are driving the proliferation in these cells. The opposite phenotype, with weak Ki67 but strong p-AKT/p-EGFR, also existed, indicating that hyperactive AKT signaling alone was insufficient in driving proliferation in these cells. More interestingly, these opposing phenotypes appeared in a sporadic pattern without obvious clustering, which suggested that they rose from intrinsic mechanisms rather than responding to extrinsic microenvironment factors. Taken together, these results underscored PACIFIC’s ability to resolve cellular heterogeneity and spatial features in FFPE samples.

### Multicolor PACIFIC

**Figure 3:**
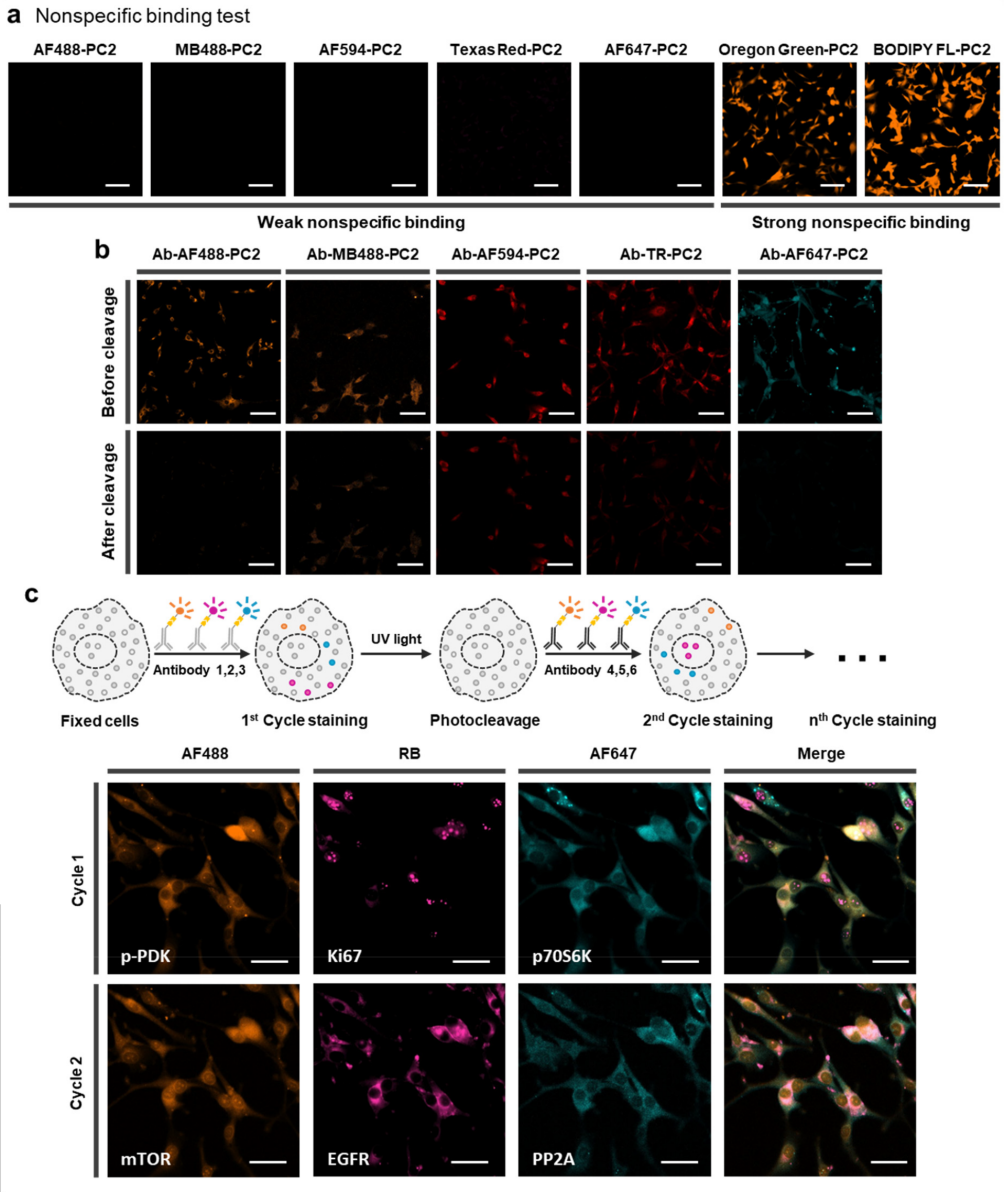
Multicolor expanding of PACIFIC. (a) Confocal images showing the nonspecific binding test results of seven commercially available fluorophores linked with PC2. Scale bar, 50 μm. (b) Five dye-PC2 linkers were conjugated to the actin antibodies, and their photoactivities were evaluated. Scale bar, 50 μm. (c) Multicolor immunofluorescence was performed to target three proteins simultaneously in U87 cells. After UV cleavage, the second round of immunostaining was performed to label another three protein targets. Scale bar, 25 μm.

We sought to increase the throughput of PACIFIC by implementing multicolor imaging. This task requires establishing a panel of fluorophores that are compatible with the PACIFIC pipeline. To this end, we conjugated seven commercially available fluorophores to the PC2 linker. We first tested the nonspecific binding tendency of these dye-PC2 linkers, and we found that Oregon Green and BODIPY-FL exhibited strong nonspecific binding toward fixed cells (Figures 3a, S13), rendering them unsuitable for PACIFIC. By contrast, AF488, MB488, AF594, Texas Red, and AF647 exhibited no nonspecific binding (Figure 3a). We then successfully conjugated the dye-PC2 linkers to the actin antibody (Figure S14) and evaluated their photoactivity under the described conditions. As shown in Figure 3b, although all these dye-PC2 linkers showed appreciable photoactivity, the cleavage efficiency for AF584, MB488, and Texas Red was suboptimal and inferior to that of AF488 and AF647. Henceforth, we chose AF488, RB, and AF647 for the multicolor PACIFIC.

To test the compatibility of these three dyes, we conjugated them to antibodies against p-PDK, Ki67, and p70S6K, and performed multicolor immunofluorescence on fixed U87 cells. As shown in Figure 3c, the obtained immunofluorescence signals were consistent with our expectations. For instance, Ki67 only appeared in the nucleus, and p70S6K exhibited more cytosolic distributions. We then tested if the signals in all three channels could be removed. Indeed, we achieved simultaneous photocleavage of these three dyes with >90% efficiency (Figure S17). We further showed that the second round of multicolor immunostaining was also successful (Figure 3c). This result supported the feasibility of multicolor PACIFIC.

### Single-Cell Analysis Using Multicolor PACIFIC

**Figure 4:**
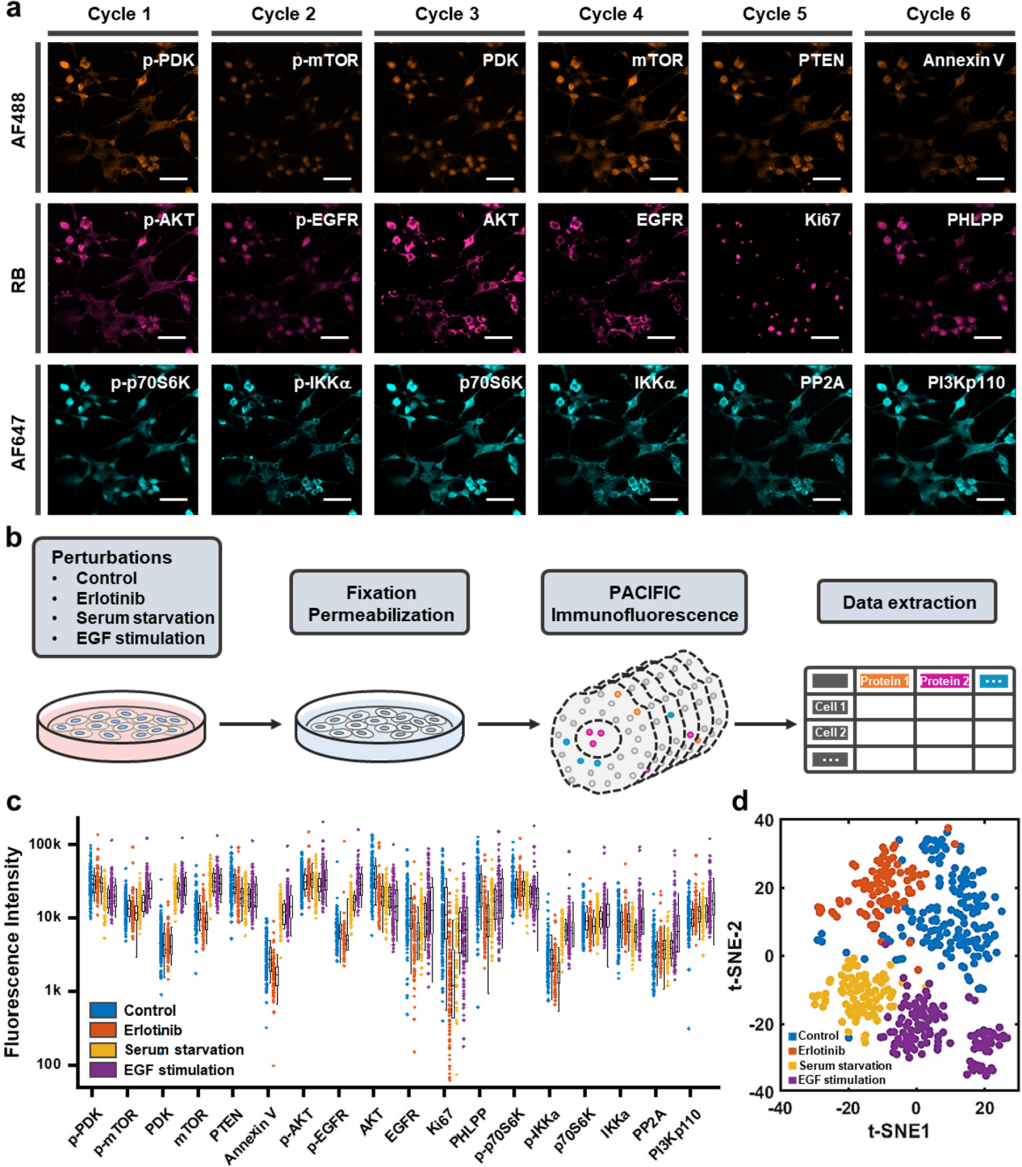
Single-cell analysis with multicolor PACIFIC. (a) Confocal images showing six cycles of PACIFIC on 18 protein targets in U87 cells. Scale bar, 50 μm. (b) Workflow of the experiment and data extraction. (c) Scatter plot of protein expression levels extracted from single cells. The boxes cover the second and the third sample quartiles, and the whiskers label the standard deviation. (d) t-SNE plot of the single-cell data set. Four clusters were observed, representing four conditions.

To expand and validate the performance of multicolor PACIFIC, we explored single-cell multiplex immunofluorescence by PACIFIC. Here, we selected 18 protein targets focusing on the EGFR-PI3K-AKT pathway, which was heavily implicated in glioblastoma biology.^43^ We prepared antibody-dye conjugates using the three dyes identified above. We performed six rounds of PACIFIC (Figures 4a, S18, S19) and extracted single-cell fluorescence intensities from each image to construct multiplex protein expression level data sets. For validation purposes, we introduced EGFR inhibition (erlotinib, 10 μM), serum starvation, and EGF stimulation (50 ng/mL) as perturbations to U87 cells and compared the resulting PACIFIC data sets (Figures 4b, S20–S22). On average, 120 cells were included in each data set. As shown in Figure 4c, PACIFIC resolved the cellular heterogeneity in protein expression levels in all four tests with consistent performance.

The PACIFIC data set captured expected signaling responses. For instance, EGFR inhibition caused a significant decrease in the p-EGFR level, while EGF treatment significantly increased the p-EGFR level. Similar responses were also observed in the cell proliferation marker (Ki67). In addition, such single-cell data sets allowed detailed analysis of analyte–analyte correlations. For example, the strong correlation between p-AKT and p-mTOR diminished upon EGFR inhibition and serum starvation but was rescued by EGF stimulation (Figure S23). By contrast, the strong correlation between p-AKT and p-EGFR was only inhibited by erlotinib treatment but unaffected by serum starvation (Figure S24). These observations were consistent with U87 biology and aligned well with our previous studies.^44,45^ From a global perspective, these differences also led to well-separated populations in the t-distributed stochastic neighbor embedding (t-SNE) analysis results (Figure 4d). Collectively, these results further showcased the capability of multicolor PACIFIC.

### Combining PACIFIC with Live-Cell Tracking to Study Cell Motility

**Figure 5:**
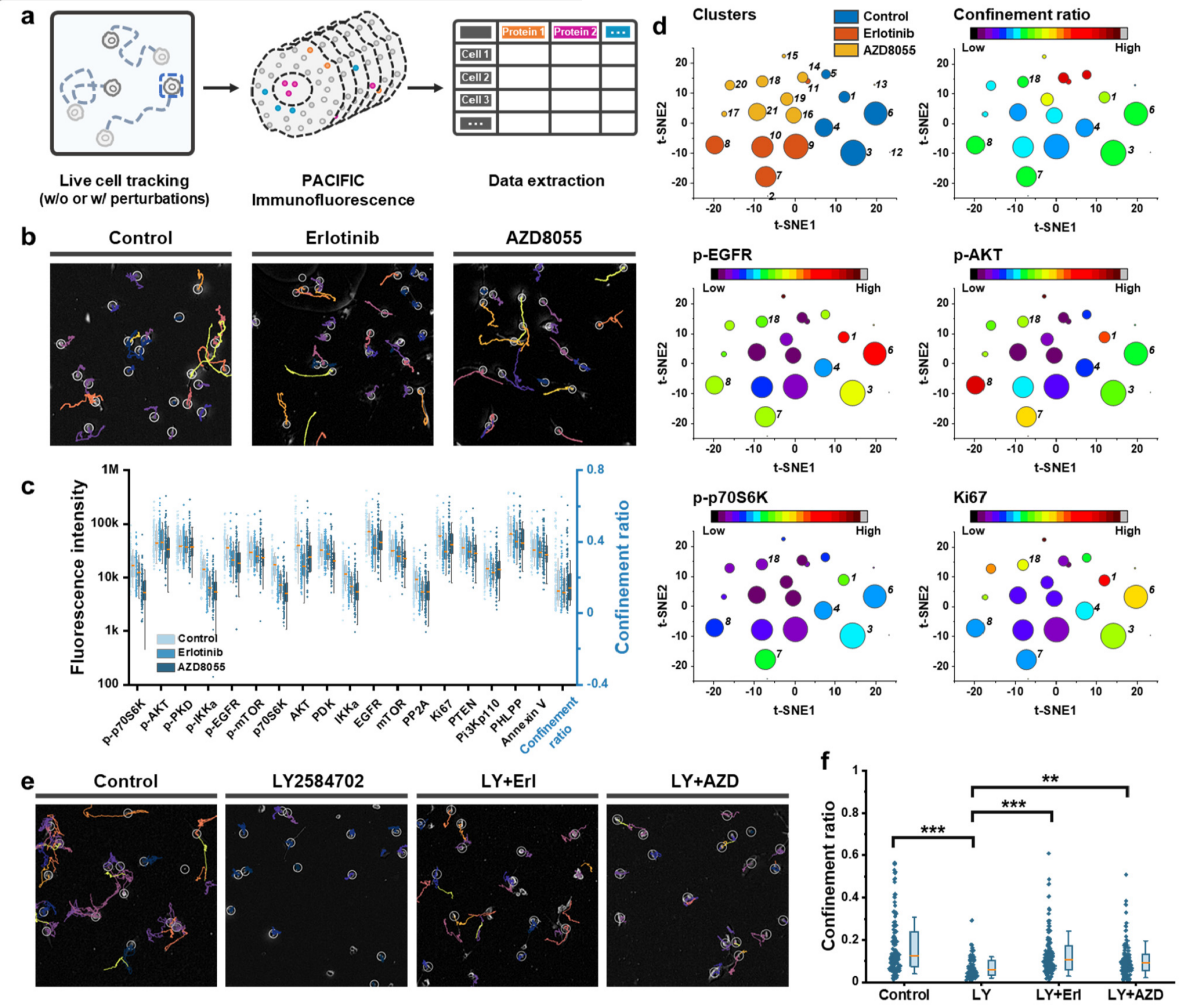
Cell motility study combining live-cell tracking with PACIFIC. (a) Workflow of the experiment. (b) Cell movement trajectories of living cells without perturbation, with EGFR inhibition (erlotinib), and with AKT inhibition (AZD8055). (c) Scatter plot of protein expression levels and the confinement ratio extracted from the single cells. (d) t-SNE plot of the single-cell data set followed by nearest neighbor clustering to partition the single-cell data into phenotypical subpopulations. (e) Cell movement trajectories of living cells without perturbation, with p70S6K inhibition (LY2584702), and combinations of LY2584702 with EGFR or AKT inhibitors. (f) Scatter plot of confinement ratio extracted from live-cell tracking. (**, *p* < 0.01; ***, *p* < 0.001).

Because PACIFIC works on fixed cells without the need for dissociation, it is possible to link other types of cell analysis with PACIFIC results. Here, we set to demonstrate combining PACIFIC with live-cell tracking. We cultured U87 cells in a Petri dish and recorded cell movements through live-cell imaging for 16 h, followed by PACIFIC analysis targeting the EGFR-PI3K-AKT pathway (Figure 5a). Our goals here were to test if the EGFR-PI3K-AKT pathway was involved in affecting U87 cell motility and identify critical proteins implicated in this process. To better understand the system, we introduced erlotinib (EGFR inhibitor) and AZD8055 (AKT inhibitor) as perturbations and focused on the confinement ratio as the metric for motility^46^ (Figure S25). TrackMate^47,48^ from Fiji was used to track the trajectories of the cell movement and obtain the confinement ratio (Figure 5b). After live-cell imaging, cells were fixed and quantified by PACIFIC analysis on 18 protein targets. As expected, both EGFR and AKT inhibition led to obvious changes in many analyte levels (Figure 5c), consistent with our results above. Interestingly, these drug treatments did not affect the average confinement ratio levels despite eliciting drastic differences in signaling activities. This finding prompted us further to analyze the interplay between oncogenic signaling and cell motility.

We leveraged more statistical analysis tools to further dissect the cellular heterogeneity and assess how each analyte contributed to the global data structure. We performed t-SNE (Figure S26) to reduce the data set dimension, followed by nearest neighbor clustering to partition the single-cell data into phenotypical subpopulations.^49^ This pipeline resolved 21 distinct clusters across three samples, with minimal overlap between the clusters (Figures 5d, S27). We found that the AZD8055 treatment significantly increased the cellular heterogeneity of the sample, evidenced by a higher number of clusters with smaller sizes, while the erlotinib treatment did not elicit such a change. This result indicated that although these two drugs acted on the same oncogenic signaling axis, their effects on U87 cells were drastically different, consistent with our previous observations.^50^ We also found that the confinement ratio was decoupled from all the other analytes on a global scale. For instance, clusters 3, 6, 7, 8, and 18 exhibited similar confinement ratios, but this similarity was not recapitulated in other analytes (Figures 5d, S27).

Interestingly, a closer evaluation of the clusters revealed several sample-dependent patterns. In the control sample, the levels of p-p70S6K and the confinement ratio exhibited a strong alignment (Figure 5d, clusters 1, 3, 4, and 6), suggesting that p-p70S6K could affect cell motility. These relationships diminished in the drug-treated samples, indicating that the relationship between p-p70S6K and cell motility may be amenable to EGFR or AKT signaling activities. Principle component analysis of each sample also generated consistent results, validating our findings from the clustering analysis (Figure S28).

These results above prompted us to generate two hypotheses: first, inhibiting p-p70S6K could lead to decreased cell motility, characterized by smaller confinement ratios; second, inhibiting EGFR and AKT signaling would antagonize the effect of p-p70S6K inhibition. To test our hypotheses, we incubated U87 cells with LY2584702 (a p70S6K inhibitor), with and without erlotinib and AZD8055, and we tracked cell movements for 16 h (Figure 5e). Indeed, p70S6K inhibition significantly decreased cell motility (Figure 5f), consistent with our predictions. More importantly, combinations of LY2584702 with erlotinib or AZD8055 obliterated the effects of LY2584702, which strongly supported our hypothesis.

## Discussion

Immunofluorescence is an indispensable tool for analyzing protein expression levels in biomedical research. Traditional IF workflows have limited multiplexity, providing limited sample information. To address this issue, approaches such as immunoSABER,^26^ chemically cleavable fluorophores,^25^ and SAFE scission^22^ were developed and successfully implemented in various studies. Nevertheless, all existing methods have their limitations, which call for additional methods to expand the repertoire of multiplex IF. The PACIFIC method reported here provides a new strategy to achieve multiplexed protein analysis, complementing existing methods and allowing integration with other types of analysis.

Compared with other multiplex IF strategies, PACIFIC has several prominent features. First, it does not introduce quencher molecules, which enables the analysis of both the expression and post-translational modification levels of the same protein. Second, it does not involve DNA-barcoding and oligomer design; therefore only needs a small reagent inventory for antibody labeling. Third, the dye-PC linker is significantly smaller than the antibody, allowing facile and quick separation via one round of spin column and minimizing spatial crowding problems. Fourth, the synthesis of the dye-PC linker does not require sophisticated organic synthesis skills, which promises simple adaptations. On the other hand, the performance of PACIFIC heavily depends on antibody availability and quality, which is a universal limitation of all IF strategies.

The current PACIFIC design also has a clear path for improvement. For instance, although ANP is cheap and its conjugation is simple, it does not have the best photoactivity. Introducing other photoactive motifs may greatly improve the cleavage efficiency and, hence, the overall performance of PACIFIC. Similarly, expanding the panel of compatible fluorophores and implementing more sophisticated microscopy methods, such as linear unmixing,^51^ will further boost the throughput and multiplexity.

The single-cell studies presented here demonstrated the application of PACIFIC, and also provided some interesting insights on U87 cell motility. As a glioblastoma cell line, U87 cells are known to exhibit high motility.^52^ Our results revealed a strong interplay between the EGFR-PI3K-AKT pathway and cell motility, and we identified p70S6K as a critical regulator. We showed that inhibiting p70S6K could suppress U87 cell motility, but combining it with targeted inhibition against EGFR and AKT led to antagonistic effects. Although the generalizability of our findings requires further studies, our results underscored the capability of PACIFIC to aid biological discoveries.

The successful integration of PACIFIC and live-cell tracking also unveiled some potential avenues worth exploring. For example, because PACIFIC does not rely on chemical cleavage or hybridization exchange, we can use a highly focused light beam to confine the cleavage to a specific area of the sample. This feature could be especially useful when implemented in a 3D tissue sample, possibly through two-photon strategies. Similarly, because no oligonucleotide is involved, PACIFIC can integrate with other DNA-encoding-based single-cell strategies, such as fluorescence in situ hybridization (FISH), to generate more comprehensive data sets and provide richer information on biological samples.

## Methods

### Materials

Rink amide MBHA resin was purchased from Aapptec (Louisville, KY). Fmoc-protected glycine was purchased from Anaspec (Fremont, CA). Fmoc-Lys(N_3_)-OH (Az4) was purchased from Chem-Impex (Wood Dale, IL). The coupling reagent HATU was purchased from Oakwood Chemical (Estill, SC). Diisopropylethylamine (DIEA, 99.5%) was purchased from ACROS (Germany). Triisopropylsilane (TIPS) and phenylsilane (PhSiH_3_) obtained from TCI (Portland, OR). Piperidine was purchased from Alfa Aesar (Ward Hill, MA). Rhodamine B (RB) and penicillin/streptomycin (PS) wereobtained from Sigma-Aldrich (St. Louis, MO). *N*,*N*′-Dimethylformamide (DMF), dimethyl sulfoxide (DMSO), methanol (MeOH), ammonium bicarbonate, Zeba spin desalting columns, and dichloromethane (DCM) were purchased from Thermo Fisher Scientific (Waltham, MA). Fmoc-PEG5-OH (98.68%) was obtained from BroadPharm (San Diego, CA). 1× Dulbecco’s modified Eagle’s medium (DMEM), fetal bovine serum (FBS), and 0.25% trypsin (with 2.21 mM EDTA) were purchased from Corning Cellgro. AF488, MB488, AF594, and AF647 were purchased from Fluoroprobes (Scottsdale, AZ). BDP FL NHS ester was purchased from Lumiprobe (Hunt Valley, MD). Sulforhodamine 101-X succinimidyl ester (Texas Red) and difluorocarboxyfluorescein succinimidyl ester (Oregon Green) were purchased from ABP Biosciences (Rockville, MD). DBCO-NHS ester and DBCO-PEG5-NHS ester were purchased from Click Chemistry Tools (Scottsdale, AZ). UV light was supplied with a high-power UV curing LED system (Thorlabs, Newton, NJ). Erlotinib, AZD8055, and LY2584702 were purchased from SelleckChem (Houston, TX). Recombinant human EGF was purchased from R&D Systems (Minneapolis, MN).

#### PC Linker Synthesis and Purification

##### Synthesis

PC linkers were synthesized through solid phase peptide synthesis (SPPS) methods (Figures S1, S3). 200 mg of Rink amide MBHA resin (loading capacity 0.67 mmol/g) was first suspended in DMF for 2 h and then deprotected with 4-methylpiperidine (20% v/v in DMF, 5 min, 3 times) followed with DMF wash (5 times). Then 0.67 mmol of Fmoc-Lys(Alloc)–OH dissolved in 2 mL of DMF was mixed with 3.25 mL of 0.2 M HATU (0.65 mmol) and 291 μL of DIEA (1.675 mmol). The mixture was added to the resin and incubated for one hour at room temperature. The solvent was then removed, and the resin was washed with DMF (5 times). The secondary amine linker and the fluorophore (and a PEG5 linker for PC2) were added following similar SPPS steps. Then the side chain of the lysine was deprotected from Alloc by the incubation with a mixture of Pd(PPh_3_)_4_ (13 mg, 11 μmol), PhSiH3 (111 μL, 1.3 mmol) in DCM (2 mL) at room temperature for 2 hours. The resulting solution was removed, and the resin was washed with the chelating solution (sodium diethyldithiocarbamate (5% w/v), DIEA (5% v/v) in DMF) 5 times, and DMF 5 times. The ANP, glycine, and the Az4 were connect to the side chain of the lysine residue following similar SPPS steps.

##### Cleavage and Purification

The resin was first washed with DCM and air-dried. The PC liners were cleaved from the resin in a TFA cleavage solution (TFA:TIPS:ddH_2_O; 95:2.5:2.5) for 2 hours. The mixture was filtered to remove the resin, and the supernatant was added to cold ethyl ether. The precipitated crude products were purified by reverse-phase HPLC (0.1% TFA in H_2_O; 0.1% TFA in acetonitrile), and the final products were confirmed using MALDI-TOF MS (AB SCIEX TOF/TOF 5800; Framingham, MA).

#### Antibody Modification

##### Antibodies

The antibodies were all purchased in carrier-free form from established vendors and stored following manufacturer recommendations. Antibodies used for PACIFIC imaging were listed in Table S1.

##### Antibody Modification with PACIFIC Linkers

The concentrations of the antibodies were prepared at 1 mg/mL in 1× PBS-0.1 M bicarbonate buffer (pH 8.4). Zeba spin columns (7k) were used for buffer exchange. After buffer exchange, the concentrations of the antibodies were validated by Nanodrop (A280). On average, the recovery of the antibodies was around 95%. Then the antibody solution was incubated with a 15-fold molar excess of the DBCO-PEG5-NHS ester (dissolved in DMSO) for 1 h at room temperature. The percentage of DMSO in the whole conjugation reaction was controlled to be less than 5%. After one hour, the reaction solution was loaded onto another 7k Zeba column (equilibrated with 1x PBS buffer) to remove the unreacted DBCO molecules. The antibody-DBCO complex solution was then incubated with a 20-fold molar excess of the PC linkers at 4 °C overnight. After the overnight reaction, the PC linker-conjugated antibodies were purified with a Zeba column to remove free PC liners. After purification, the absorbance spectrum of the conjugated antibodies was measured using a Synergy H1 microplate plate reader. The degree of labeling (DOL) was calculated based on the known extinction coefficients of the antibodies and dyes. The conjugated antibodies were stored in the dark at 4 °C in PBS with 0.02% sodium azide for future use.

#### Cell Culture Methods

##### Cell Line

The human glioblastoma cell line (U87) was purchased from the American Type Culture Collection (Manassas, VA). U87 cells were cultured in Dulbecco’s modified Eagle’s medium (DMEM) supplemented with 10% heat-inactivated fetal bovine serum and 100 U/mL penicillin/streptomycin in a humidified 5% CO_2_ (v/v) incubator at 37 °C. All the experiments used the same culture medium if not otherwise stated.

#### PACIFIC Imaging and UV Cleavage

##### Cell Preparation

U87 cells were first seeded in a 96-well plate or a 35 mm Petri dish overnight. Then the cells were fixed with ice-cold methanol for 15 min. Gently wash the cells with PBS (5 min, 3 times). Block the cells with blocking buffer (5% normal goat serum and 0.3% Triton X-100 in PBS) at room temperature for one hour. Aspirate the blocking buffer, and incubate the cells with the modified antibody solution according to the manufacture’s suggestion. Then gently wash the cells with PBS (5 min, 3 times), and it is ready for confocal imaging.

##### FFPE Tissue Preparation

Deparaffinize and rehydrate the FFPE tissue slides (US BioMax Inc, Derwood, MD) through the following sequence: (a) xylene, 5 min, 3 times; (b) 100% ethanol, 5 min; (c) 95% ethanol, 5 min; (d) 70% ethanol, 5 min; (e) 50% ethanol, 5 min; (f) deionized water, 5 min. Block the tissue with blocking buffer (5% normal goat serum and 0.3% Triton X-100 in PBS) at room temperature for one hour. Aspirate the blocking buffer and incubate the tissue with the modified antibody solution according to the manufacturer’s suggestion. Then gently wash the cells with PBS (5 min, 3 times), and they are ready for confocal imaging.

##### Fluorescence Imaging and Analysis

The immunofluorescence images were collected using a Zeiss 880 inverted confocal laser scanning microscope (Carl Zeiss MicroImaging GmbH, Jena, Germany). The laser power and detection gain were tuned for an optimized signal-to-noise ratio and kept the same for different cycles. Fluorescence intensities were extracted using Fiji software.

##### UV Cleavage

After confocal imaging, exchange the PBS buffer with Argon-purged methanol. Irradiate the sample with 365 nm UV light. The power was set to 90%. After that, wash the cells with PBS (5 min, 3 times). The sample was imaged again with confocal at the same spot to quantify the cleavage efficiency. Then it was ready for the next cycle of immunofluorescence.

#### Live Cell Tracking and Motility Analysis

##### Live Cell Tracking

To prepare the cells, 35k of U87 cells were seeded in a 35 mm Petri dish and incubated overnight. On the second day, the seeding medium was replaced by 2 mL of fresh media with various drugs, and the cells were continuously monitored with an EzScope 101 live-cell imaging system (Blue-Ray Biotech, Taiwan) for 16 h at 2-min integrals inside a humidified 5% CO_2_ (v/v) incubator at 37 °C. Cell motility data were extracted from the recorded videos using TrackMate from Fiji software.

## Supporting Information Available

Control 1 live-cell tracking (AVI)
Control 2 live-cell tracking (AVI)
AZD8055 + LY2584702 live-cell tracking (AVI)
Erlotinib live-cell tracking (AVI)
AZD8055 live-cell tracking (AVI)
Erlotinib + LY2584702 live-cell tracking (AVI)
LY2584702 live-cell tracking (AVI)
Synthetic schemes and characterization data of the PC linkers, additional confocal images, additional experimental results, and statistical analysis results (PDF)

## Supplementary Material

bg3c00018_si_001.avi

bg3c00018_si_002.avi

bg3c00018_si_003.avi

bg3c00018_si_004.avi

bg3c00018_si_005.avi

bg3c00018_si_006.avi

bg3c00018_si_007.avi
