# Supplementary material for: Multiplex Protein Imaging through PACIFIC: Photoactive Immunofluorescence with Iterative Cleavage
Source: ACS Bio Med Chem Au. 2023 Apr 28;3(3):283–94. doi: 10.1021/acsbiomedchemau.3c00018 (PMC10288499; doi:10.1021/acsbiomedchemau.3c00018)
Supplement: Supplementary file 8 — bg3c00018_si_008.pdf [file bg3c00018_si_008.pdf]

Multiplex protein imaging through PACIFIC: photoactive immunofluorescence with iterative cleavage

Fei Ji[†], Moises Hur[¶], Sungwon Hur[¶], Siwen Wang[†, ‡], Priyanka Sarkar[†], Shiqun Shao[†, §], Desiree Aispuro[†, ‡], Xu Cong[†], Yanhao Hu[††], Zhonghan Li[† *], Min Xue[†, ‡ *]

Affiliations:

[†]Department of Chemistry, University of California, Riverside, Riverside, California 92521, United States

[‡]Environmental Toxicology Graduate Program, University of California, Riverside, Riverside, California 92521, United States

[§]College of Chemical and Biological Engineering, Zhejiang University, Hangzhou, Zhejiang 310027, P.R. China

[¶]Martin Luther King Jr High School, Riverside, California 92508, United States

[††]Diamond Bar High School, Diamond Bar, California 91765, United States

[*]Corresponding authors: zhonghan@ucr.edu (ZL), minxue@ucr.edu (MX)

Supporting Information

PACIFIC antibodies:

Target protein	Clone	Host species	Vendor	Catalog #	Fluorophore	Degree of labeling
EGFR	Polyclonal	Goat	R&D	AF231	Rhodamine B	3.0
p-EGFR (Y1086)	Monoclonal	Rat	R&D	MAB89671	Rhodamine B	3.2
AKT	Monoclonal	Mouse	R&D	MAB2055	Rhodamine B	2.7
p-AKT (S473)	Polyclonal	Rabbit	R&D	AF887	Rhodamine B	2.7
Ki67	Monoclonal	Rabbit	R&D	MAB7617	Rhodamine B	2.9
PHLPP	Polyclonal	Rabbit	NovusBio	NBP2-81766	Rhodamine B	2.2
PDK1	Monoclonal	Mouse	R&D	MAB864	AF488	4.7
p-PDK1 (S241)	Polyclonal	Rabbit	Cell Signaling	3061	AF488	2.8
mTOR	Monoclonal	Rat	R&D	MAB1537	AF488	6.8
p-mTOR (S2448)	Monoclonal	Rat	R&D	MAB1665	AF488	7.4
PTEN	Polyclonal	Rabbit	R&D	AF847	AF488	6.1
Annexin V	Monoclonal	Mouse	R&D	MAB3991	AF488	6.6
IKKα	Polyclonal	Sheep	R&D	AF3768	AF647	5.8
p-IKKα (S176/S180)	Monoclonal	Rat	R&D	MAB3768	AF647	6.1
p70S6K	Polyclonal	Rabbit	R&D	AF8962	AF647	5.4
p-p70S6K (T421/S424)	Polyclonal	Rabbit	R&D	AF8965	AF647	6.4
PP2A	Monoclonal	Rat	R&D	MAB1653	AF647	6.5
PI3Kp110α	Polyclonal	Rabbit	Cell Signaling	4255	AF647	5.7
β-actin	Monoclonal	Mouse	R&D	MAB8929	Rhodamine B<br>AF488<br>AF647	3.7<br>4.3<br>5.6

Table S1. PACIFIC antibodies. All the antibodies were purchased as BSA-free and validated by their manufacturers. After modification and purification, the concentrations of the antibodies were measured by Nanodrop. For PACIFIC imaging, the antibodies were diluted to the concentrations suggested by the manufacturers.

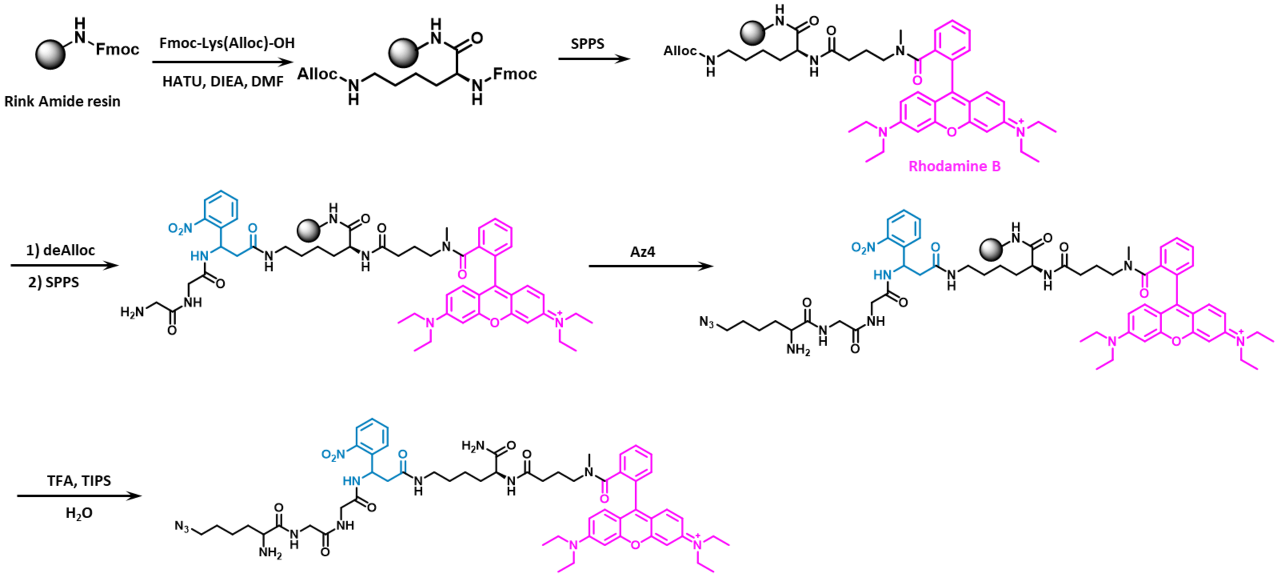

Figure S1. Synthetic scheme of RB-PC1.

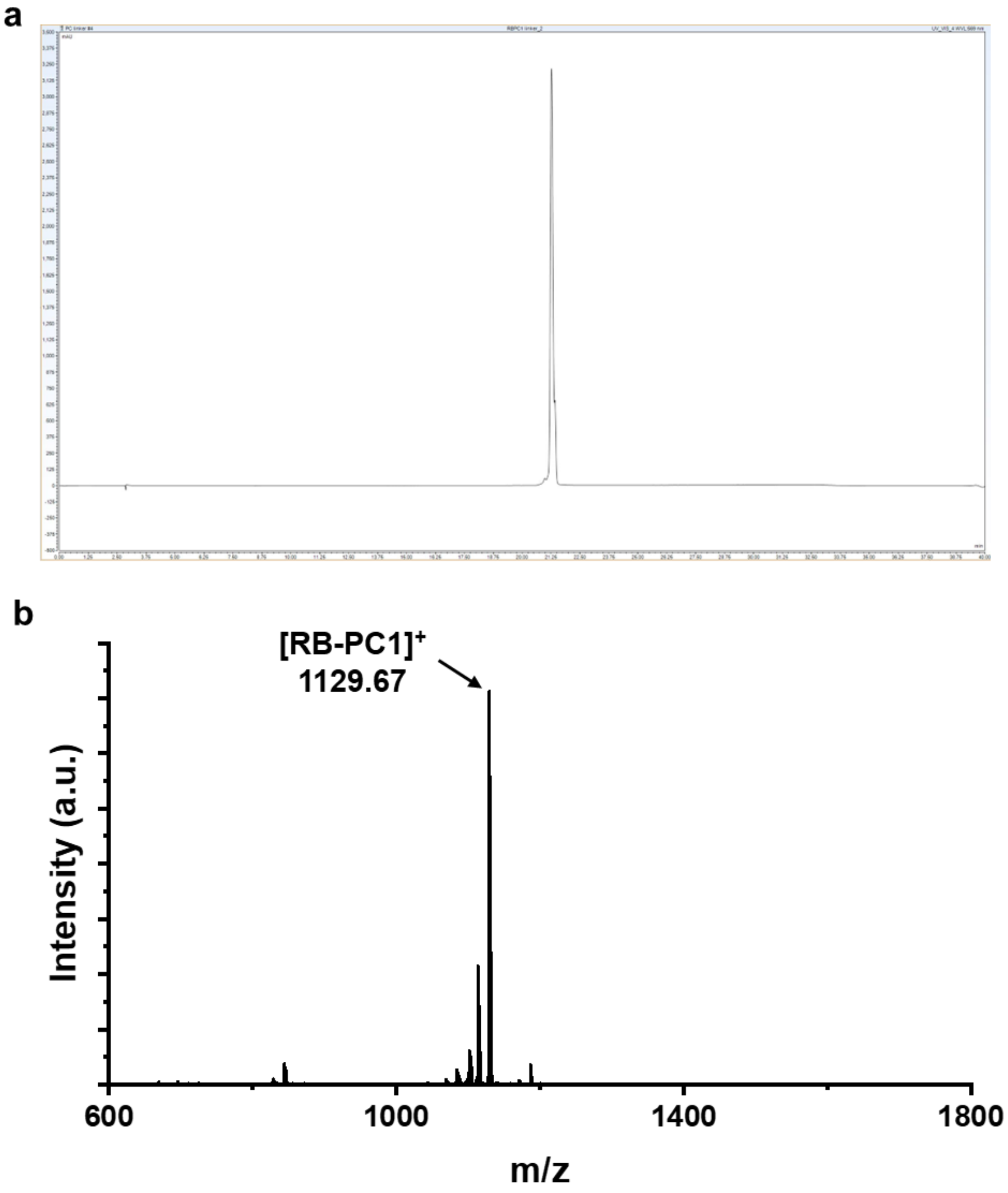

Figure S2. a. Analytical HPLC chromatogram of RB-PC1. **b.** Mass spectrum of RB-PC1 (MALDI-TOF). $[M+H]^+$ calculated 1129.59, found 1129.67.

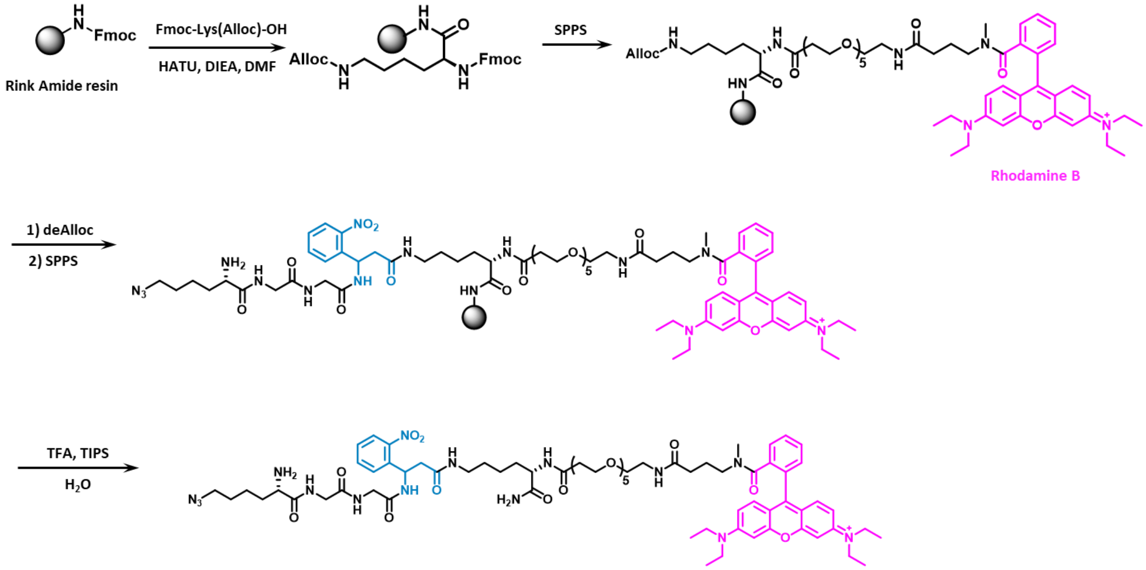

Figure S3. Synthetic scheme of RB-PC2.

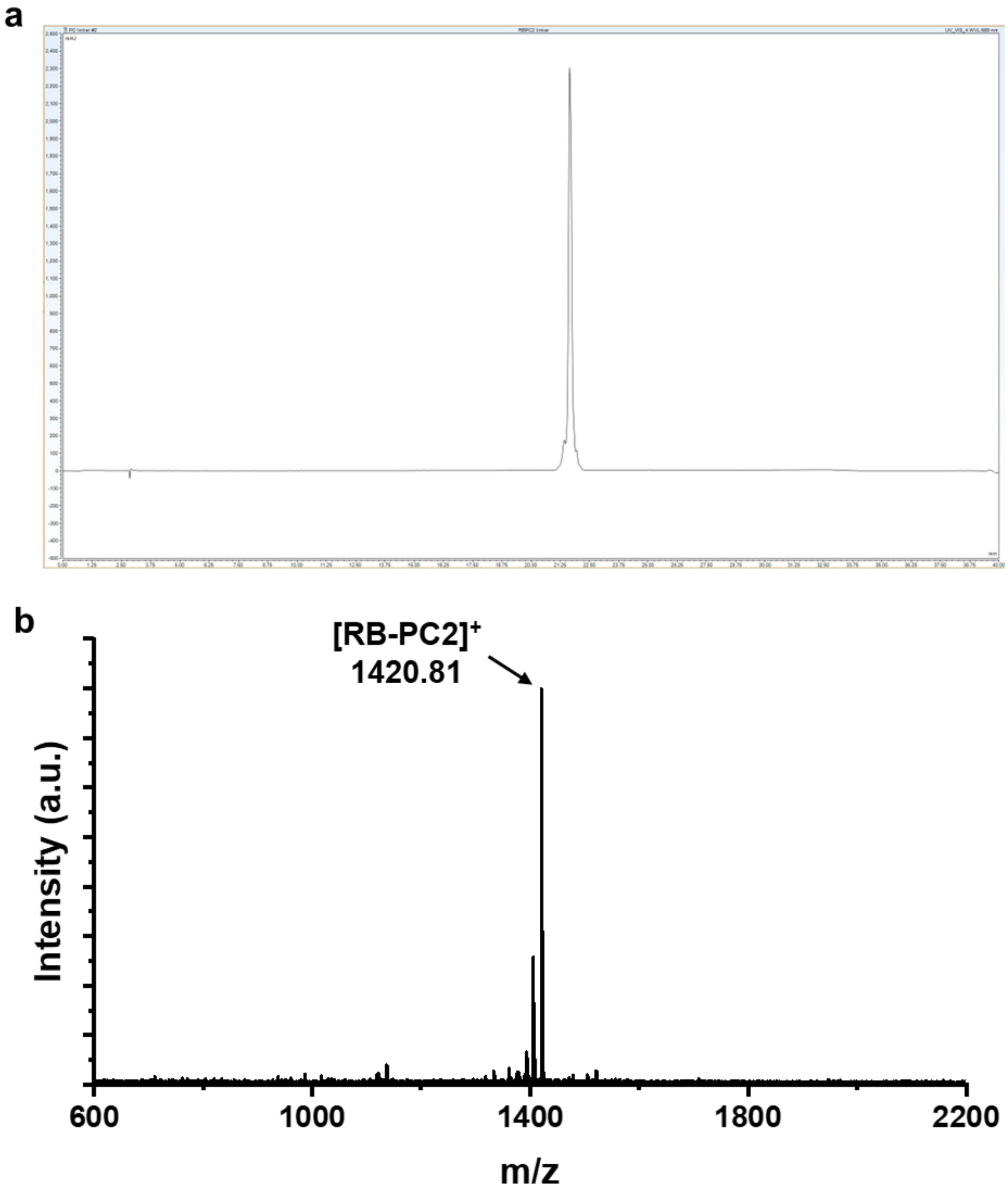

Figure S4. a. Analytical HPLC chromatogram of RB-PC2. **b.** Mass spectrum of RB-PC1 (MALDI-TOF). $[M+H]^+$ calculated 1420.76, found 1420.81.

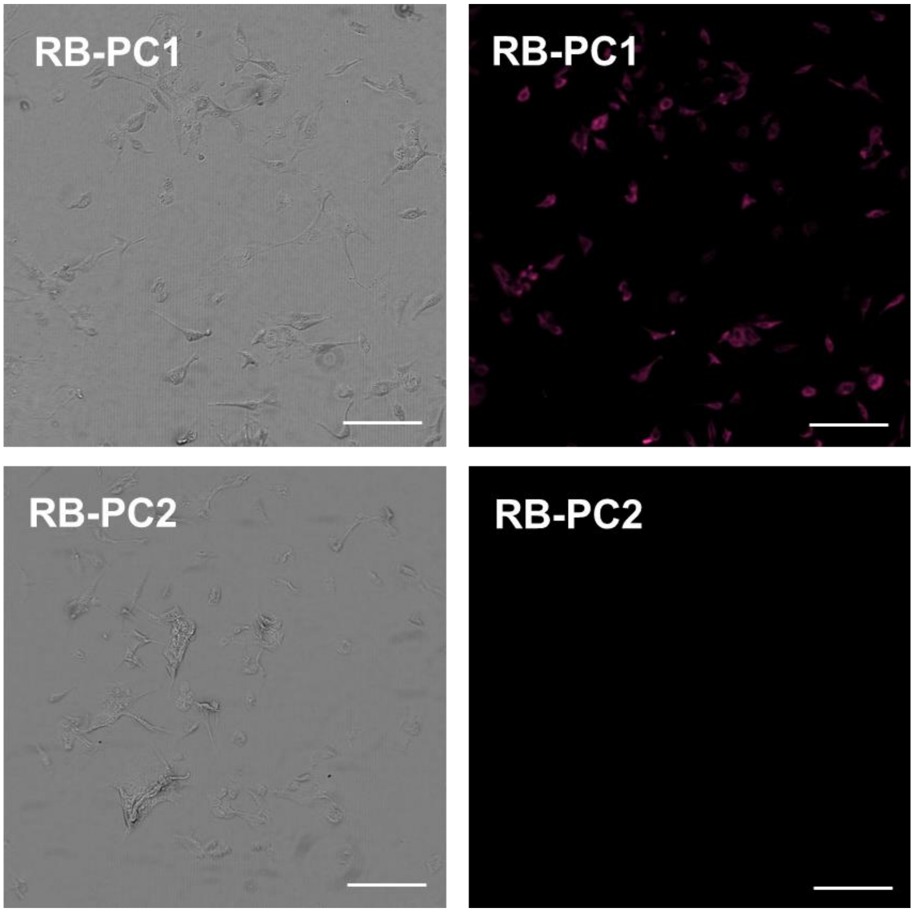

Figure S5. Confocal images showing the nonspecific binding test results of RB-PC1 and RB-PC2 on fixed U87 cells. Scale bar, 50 μm.

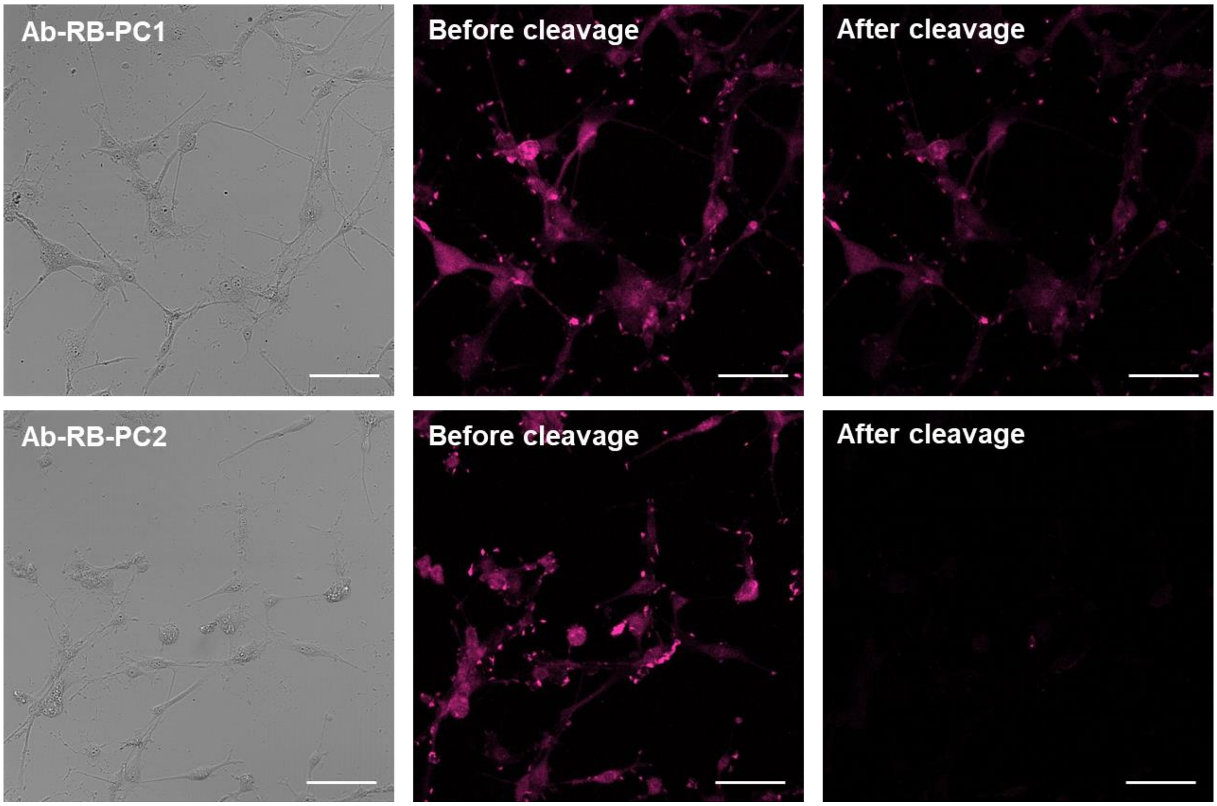

Figure S6. Confocal images showing the UV cleavage test results of Ab-RB-PC1 and Ab-RB-PC2 in methanol. Scale bar, 50 μm.

a

Equivalents of DBCO	Degree of labeling (DOL)
5	0.98
10	1.71
15	3.67
30	0.93

b

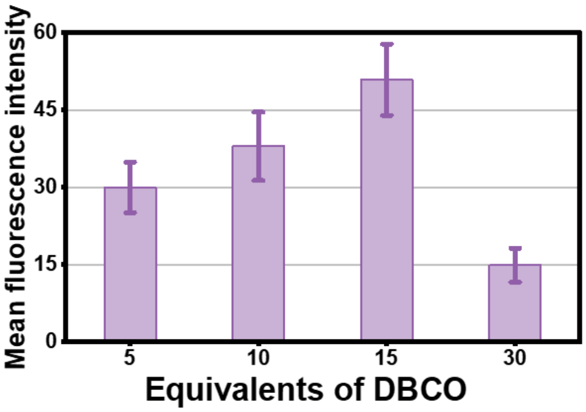

Figure S7. Optimizing the conjugation labeling. a. The calculated degree of labeling (DOL) with various equivalents of DBCO to antibody. With an increasing amount of DBCO to 30 equivalents, precipitation of antibody-DBCO complex was observed and decreased the antibody recovery yield. **b.** The mean fluorescence intensity obtained from the different equivalents of DBCO-modified antibodies. The 15-equivalent-modified antibody showed the highest signal, which was consistent with the DOL results.

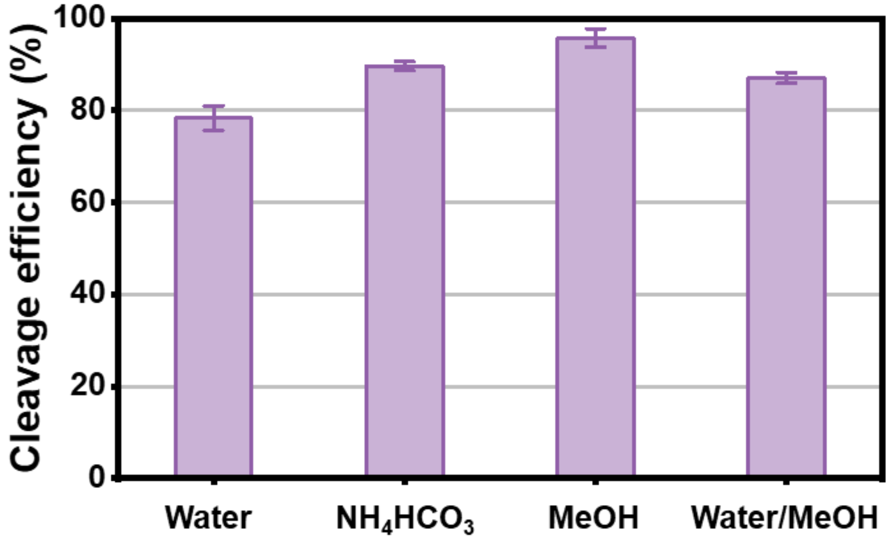

Figure S8. Cleavage efficiencies of Ab-RB-PC2 in different solvents for one hour UV cleavage.

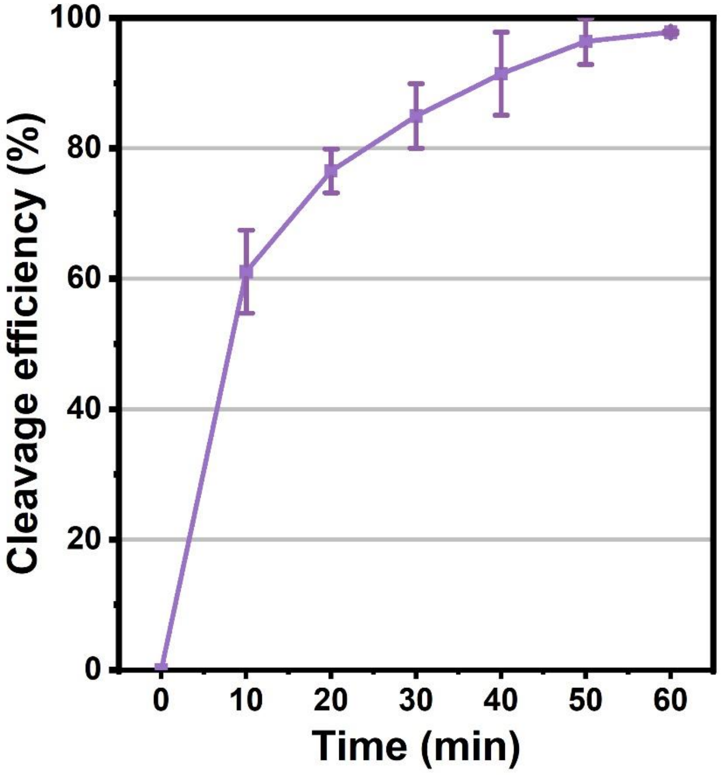

Figure S9. Time-dependent cleavage efficiency of Ab-RB-PC2 in argon-purged methanol.

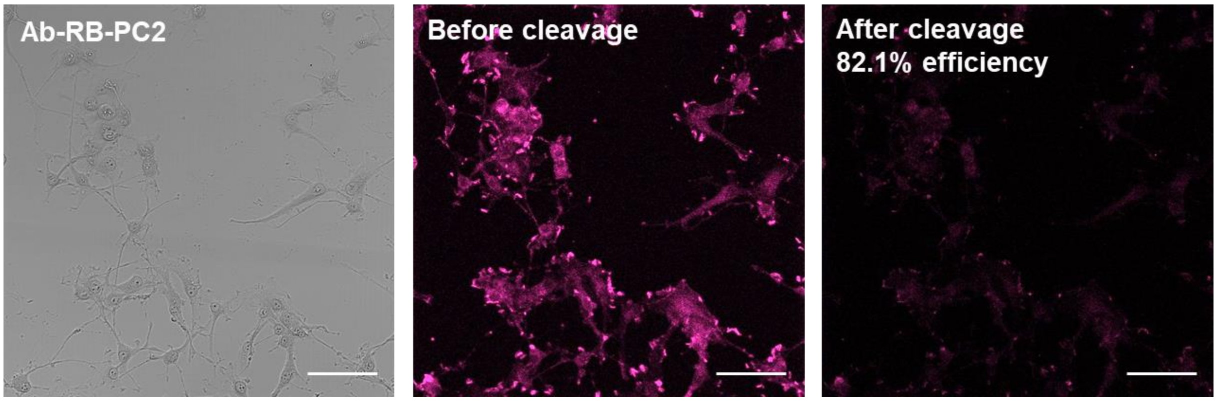

Figure S10. Confocal images showing UV cleavage results of Ab-RB-PC2 in methanol without argon purging. The cleavage efficiency was 82.1%. Scale bar, 50 μm

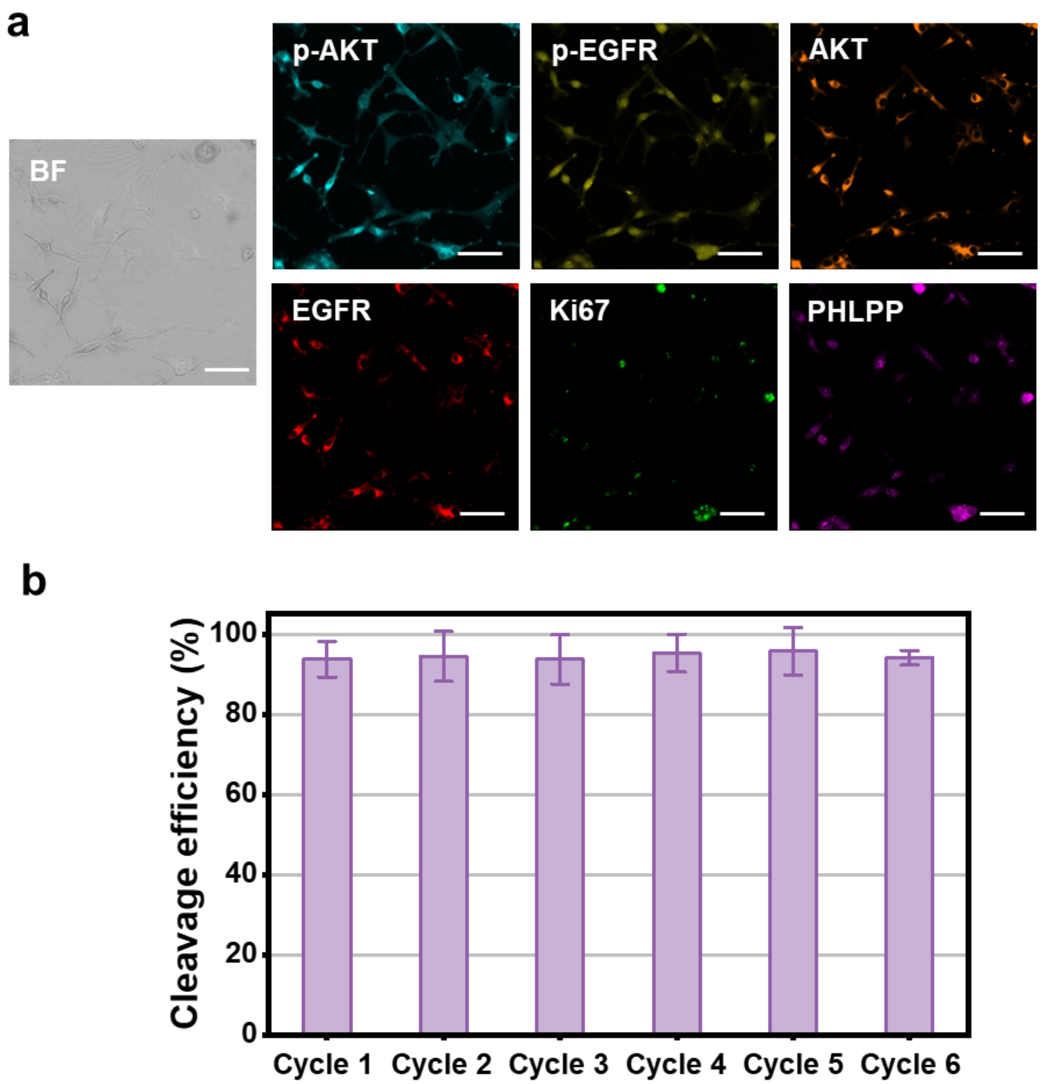

Figure S11. a. Confocal images showing the bright field and 6 cycles of immunofluorescence images at the same spot of U87 cells. Scale bar, 50 μm. **b.** Cleavage efficiencies of Ab-RB-PC2 for the 6 cycles.

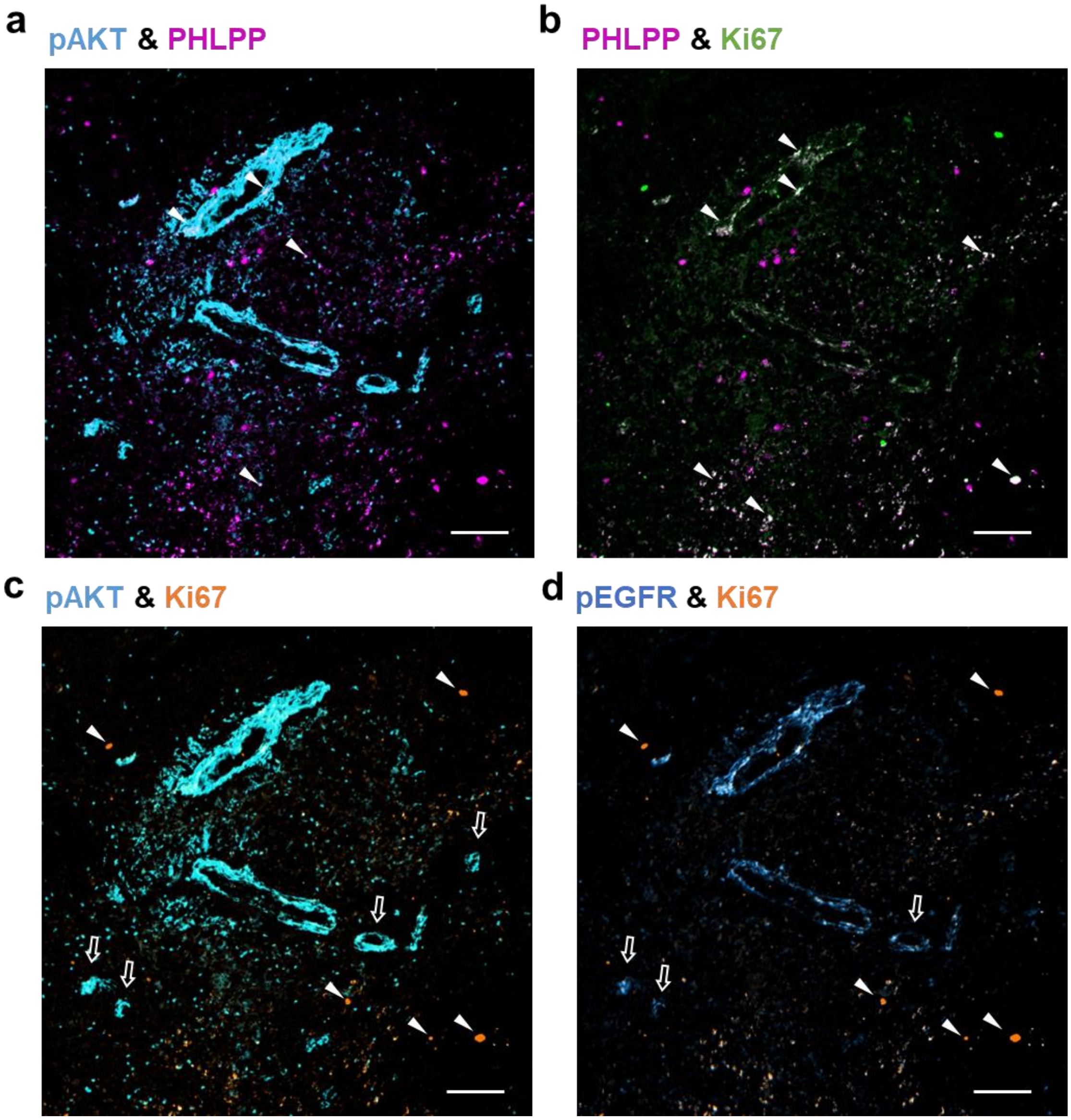

Figure S12. Merged channels of the GBM tissue sample. a. Merged channels of p-AKT and PHLPP. Triangles mark cells with strong signals of both p-AKT and PHLPP. **b.** Merged channels of PHLPP and Ki67. Triangles mark cells with strong signals of both PHLPP and Ki67. **c.** Merged channels of p-AKT and Ki67. Triangles mark cells with strong Ki67 signals but low p-AKT signals, and arrows mark cells with strong p-AKT signals but low Ki67 cells. **d.** Merged channels of p-EGFR and Ki67. Triangles mark cells with strong Ki67 signals but low p-EGFR signals, and arrows mark cells with strong p-EGFR signals but low Ki67 cells. Scale bar, 100 μm.

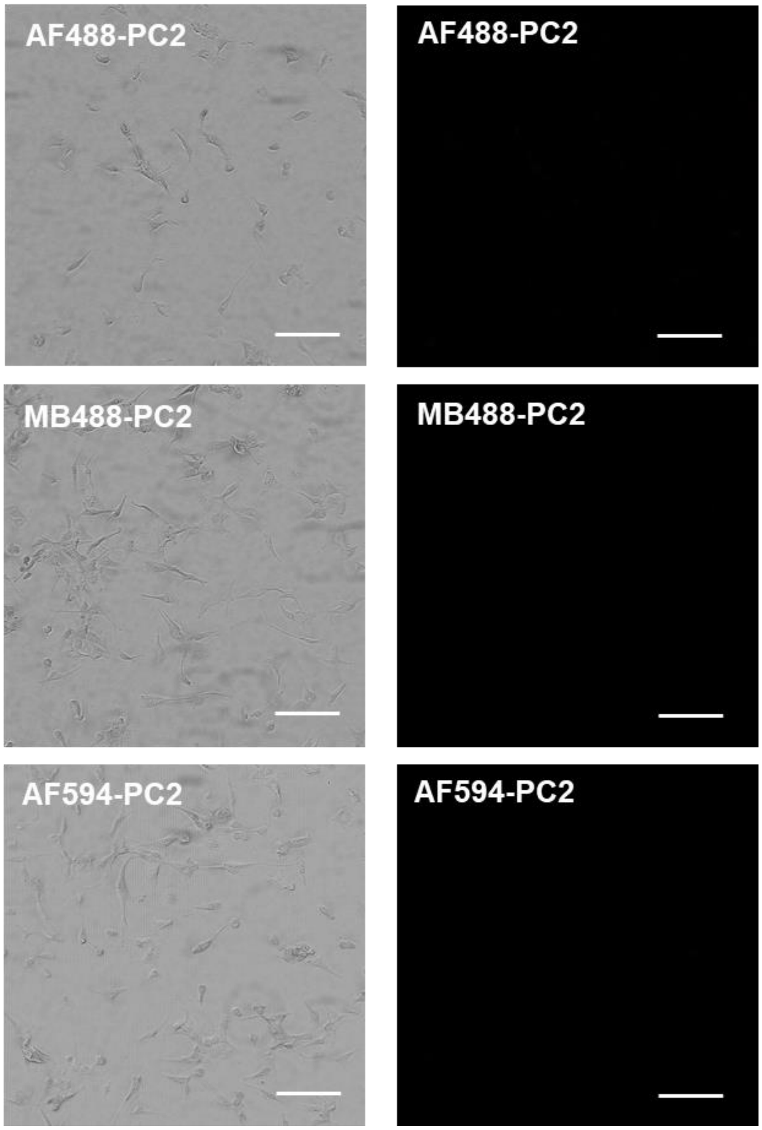

Figure S13. Confocal images showing the nonspecific binding test results of AF488-PC2, MB488-PC2, and AF594-PC2. Scale bar, 50 μm.

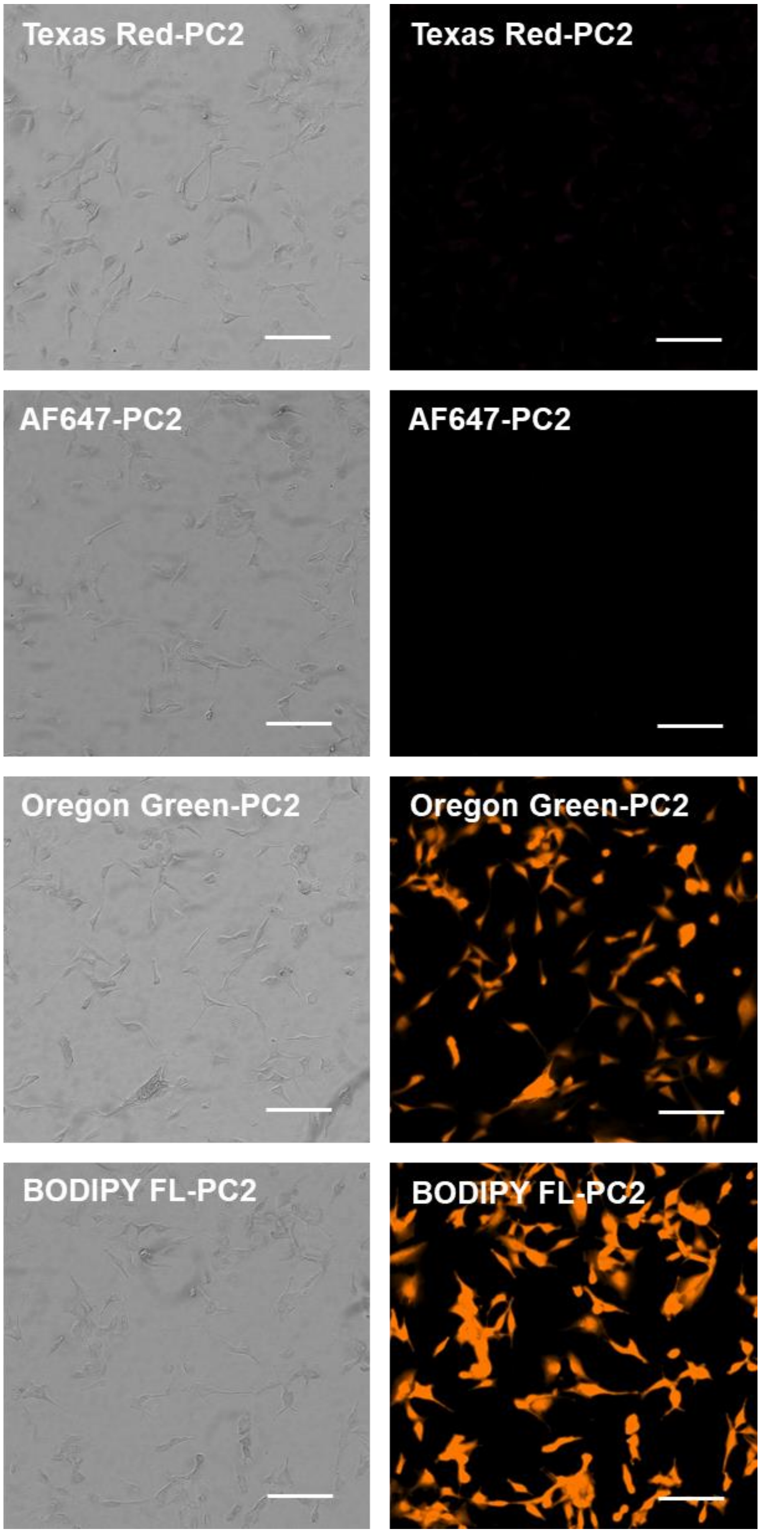

Figure S13. (continued) Confocal images showing the nonspecific binding test results of Texas Red-PC2, AF647488-PC2, and Oregon Green-PC2, and BODIPY FL-PC2. Scale bar, 50 μm.

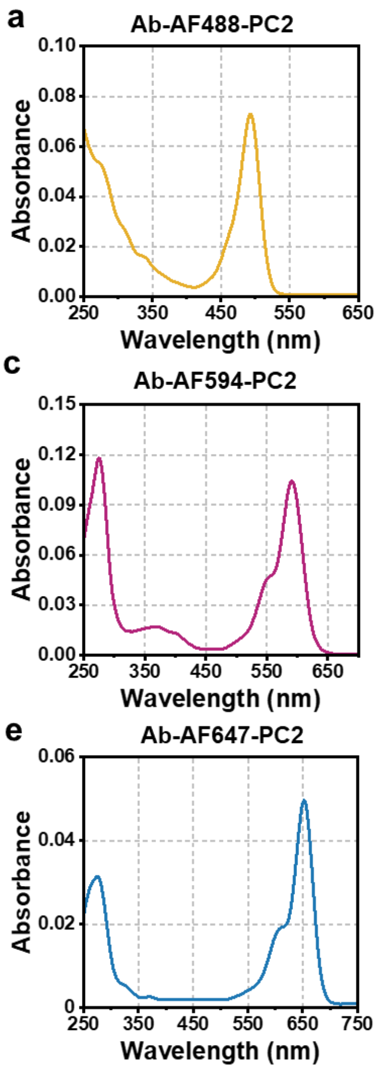

Figure S14. UV-vis absorption spectra of five Ab-dye-PC2 constructs, showing antibody absorption at ~280 nm.

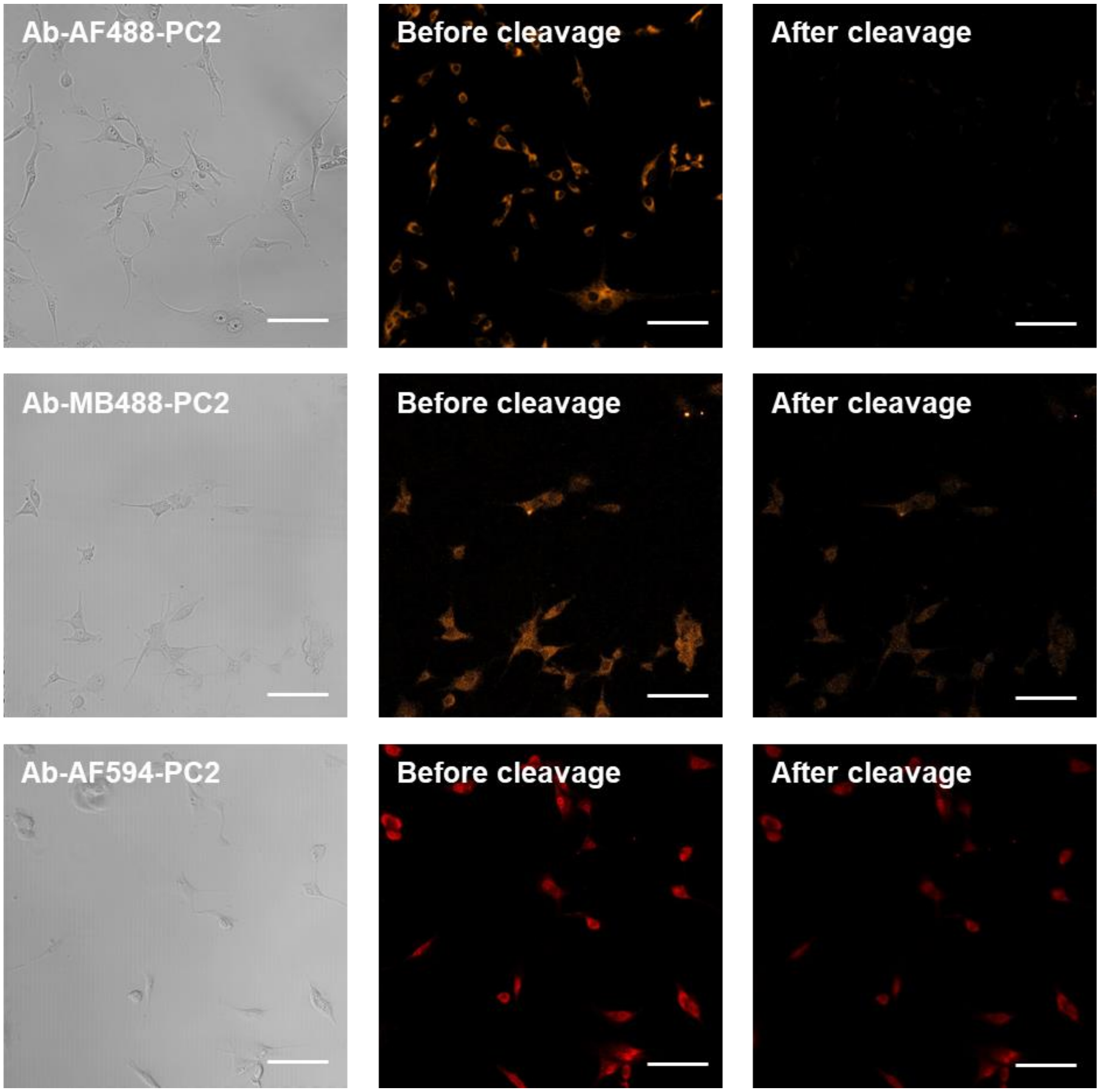

Figure S15. Confocal images showing the UV cleavage test results of Ab-AF488-PC2, Ab-MB488-PC2, and Ab-AF594-PC2. Scale bar, 50 μm.

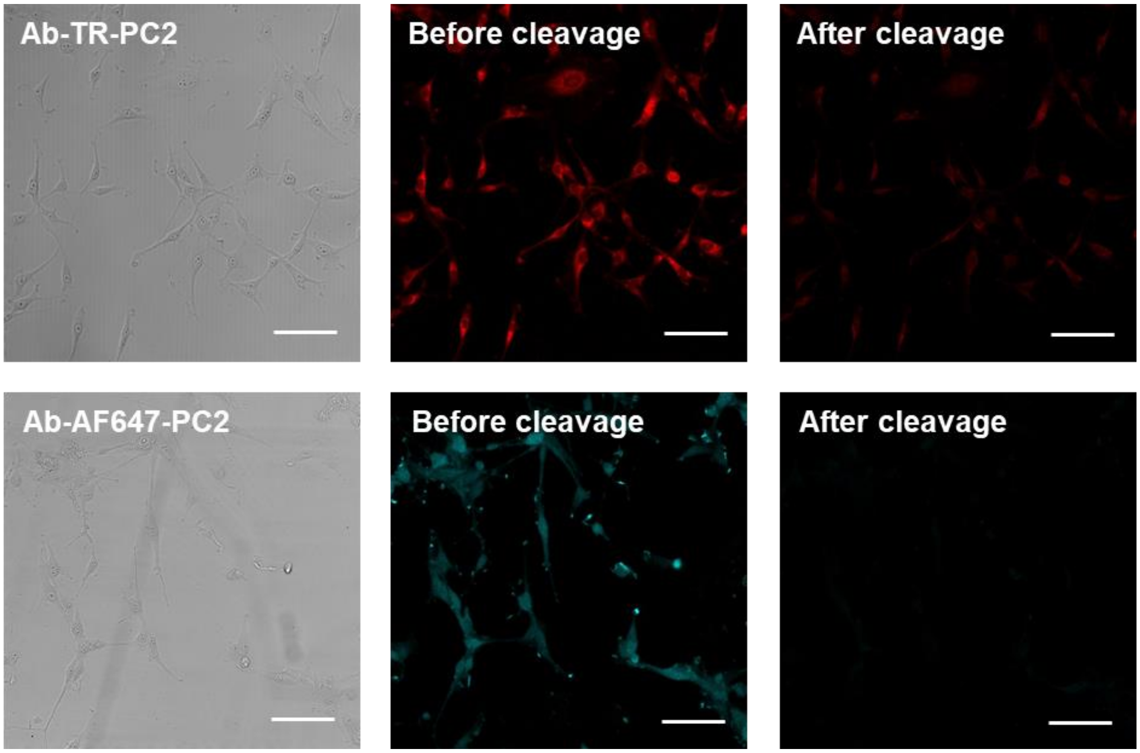

Figure S15 (continued). Confocal images showing the UV cleavage test results of Ab-TR-PC2 and Ab-AF647-PC2. Scale bar, 50 µm.

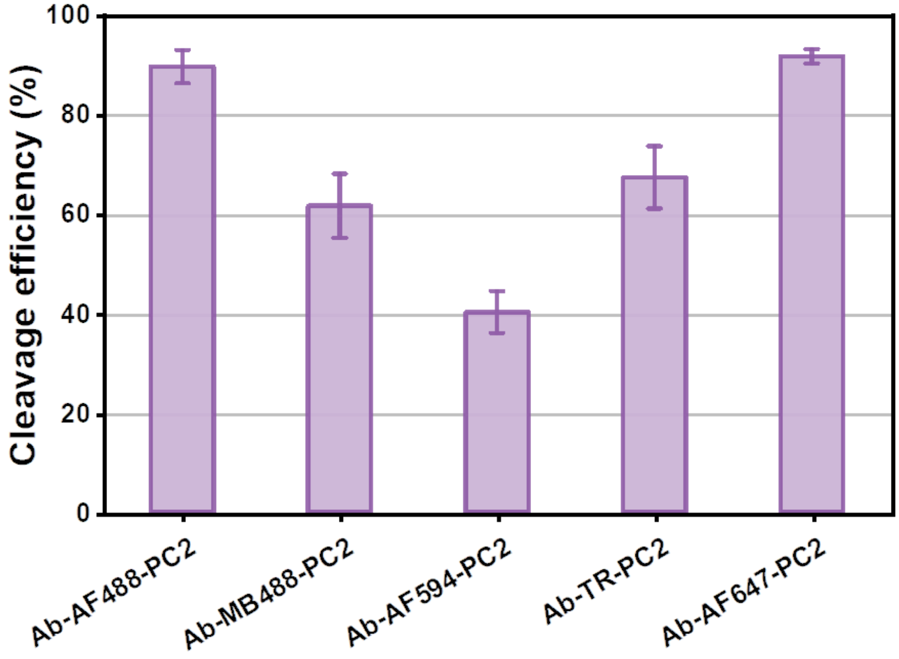

Figure S16. Summary of cleavage efficiencies of Ab-AF488-PC2, Ab-MB488-PC2, Ab-AF594-PC2, Ab-TR-PC2, and Ab-AF647-PC2. The values are 90.9%, 61.7%, 40.3%, 67.4%, and 91.8%, respectively.

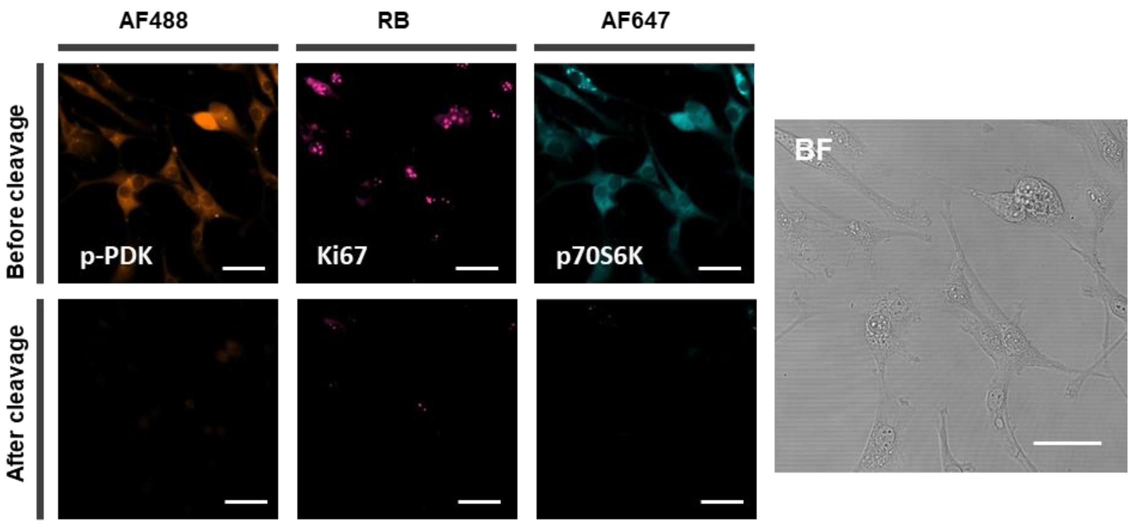

Figure S17. Confocal images showing multi-color immunofluorescence targeting three different proteins and UV cleavage afterwards.

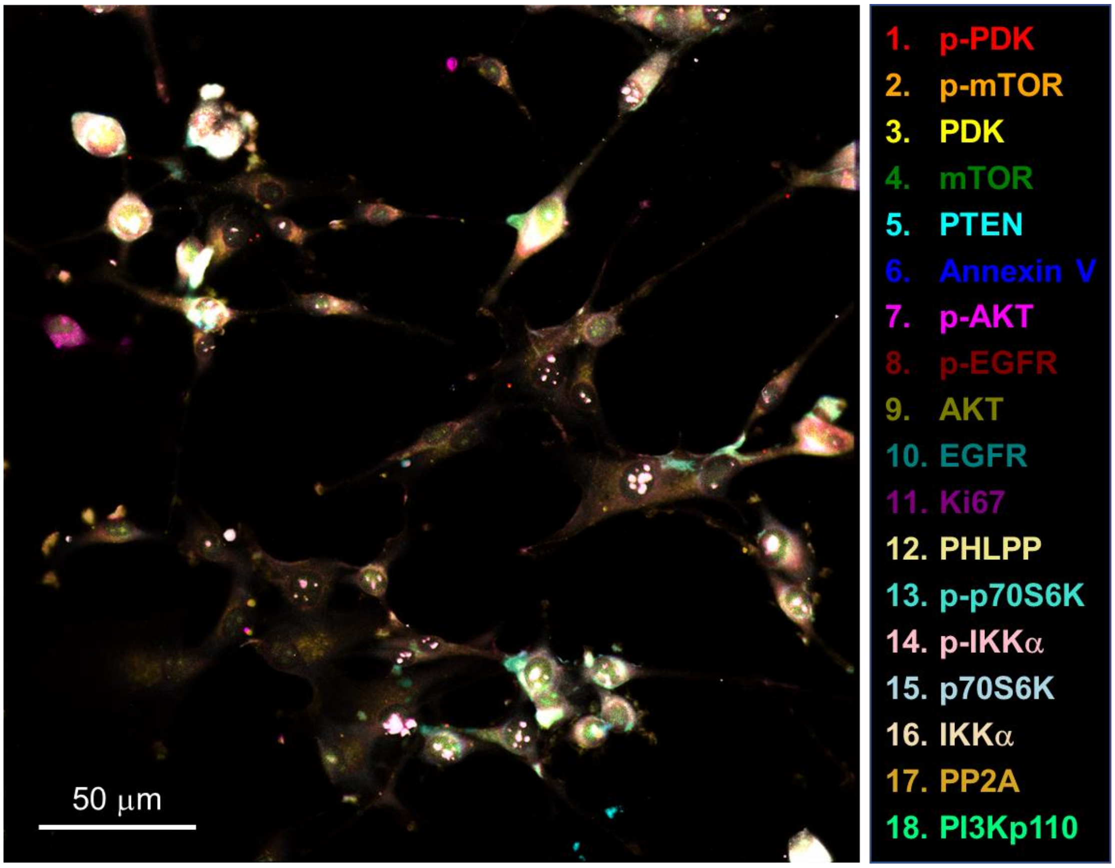

Figure S18. False color image showing 18 different protein targets in one spot. The colors are artificially selected to demonstrate the multiplexity, and the contrast of each channel is adjusted for clarity.

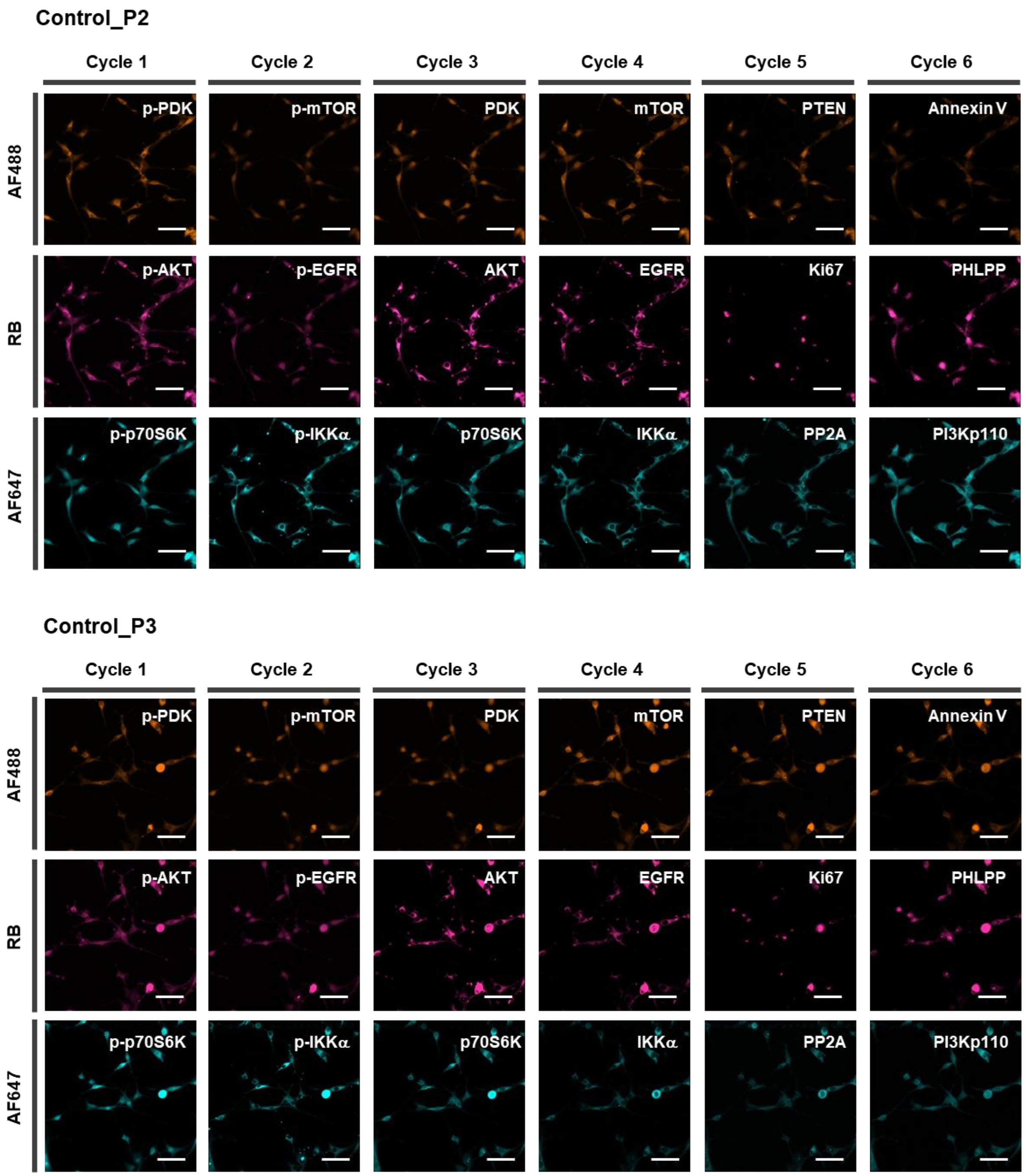

Figure S19. More representative confocal images showing six cycles of PACIFIC on 18 protein targets in control samples.

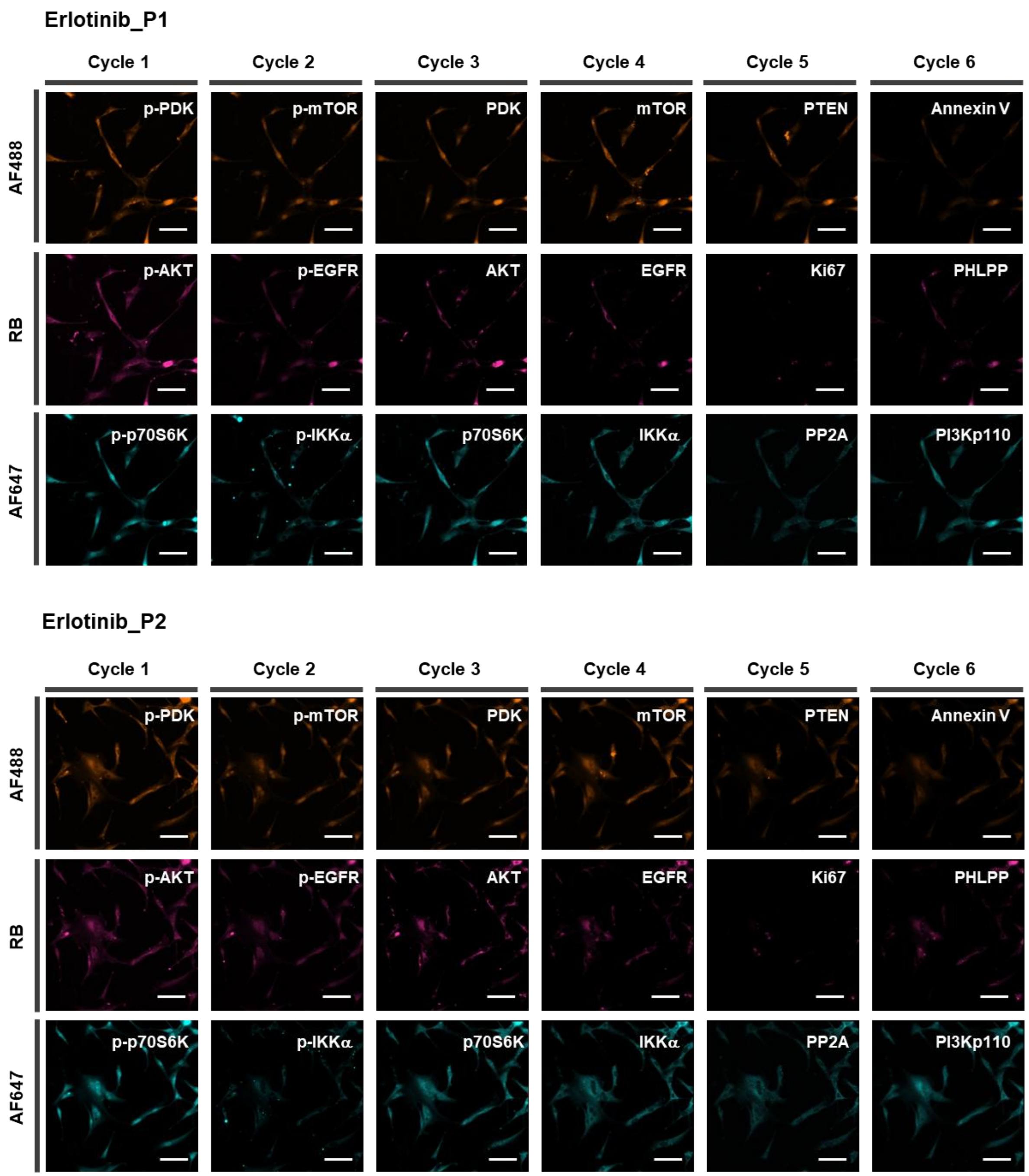

Figure S20. Representative confocal images showing six cycles of PACIFIC on 18 protein targets in erlotinib-treated samples.

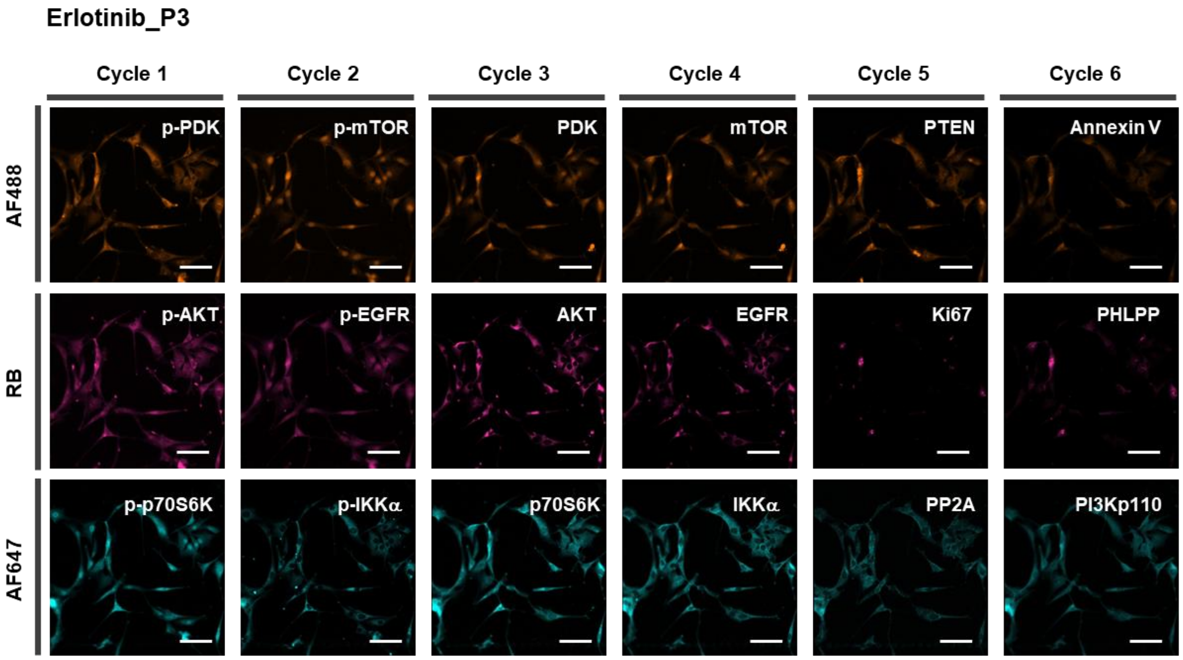

Figure S20 (continued). Representative confocal images showing six cycles of PACIFIC on 18 protein targets in erlotinib-treated samples.

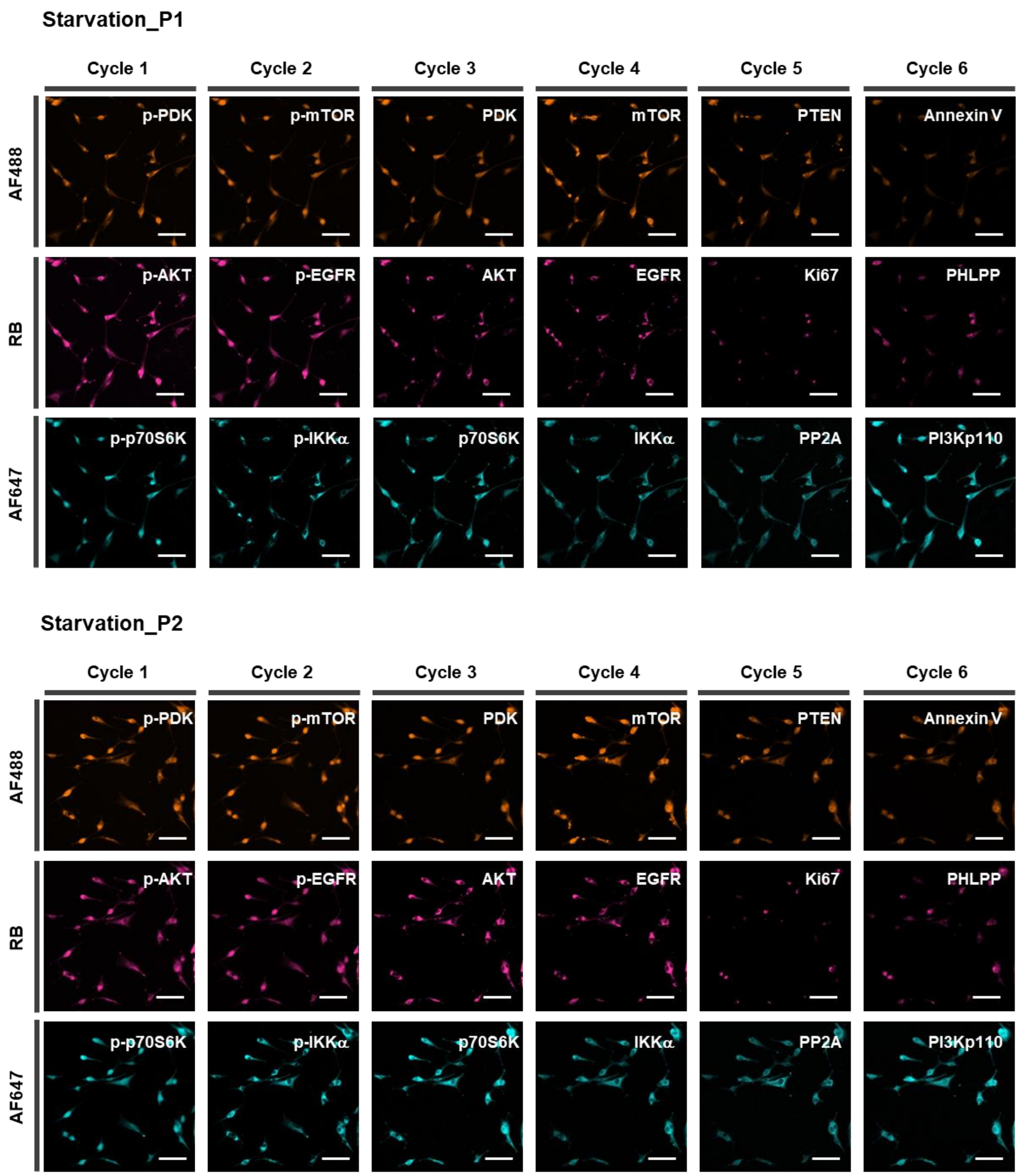

Figure S21. Representative confocal images showing six cycles of PACIFIC on 18 protein targets in serum-starved samples.

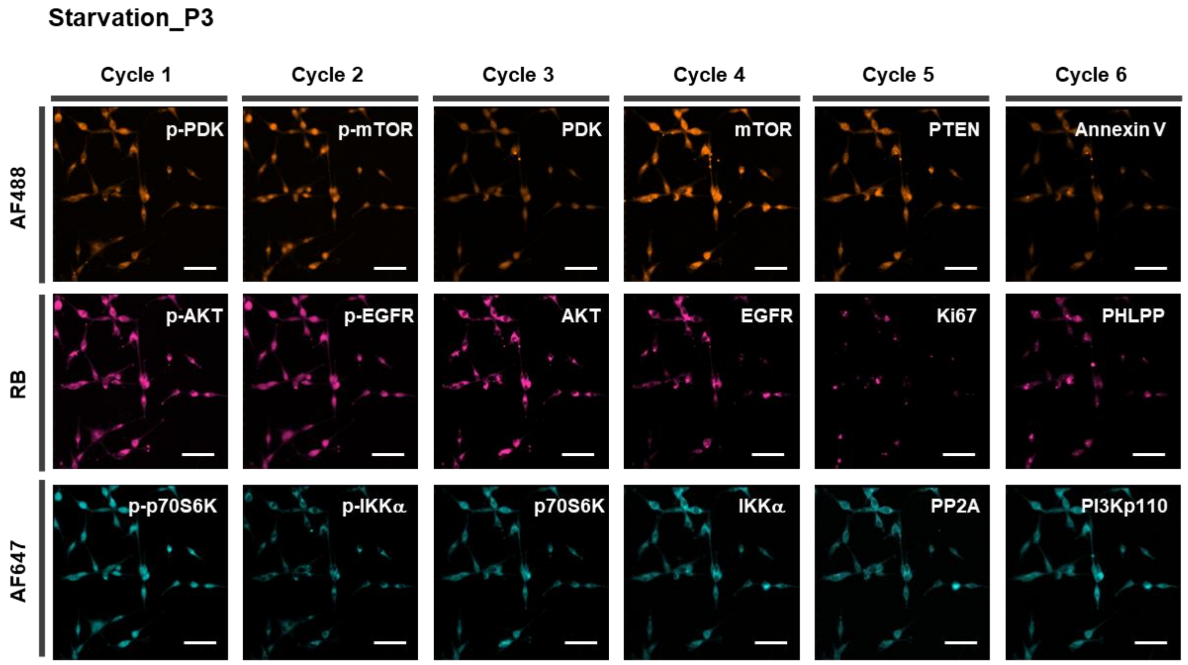

Figure S21 (continued). Representative confocal images showing six cycles of PACIFIC on 18 protein targets in serum-starved samples.

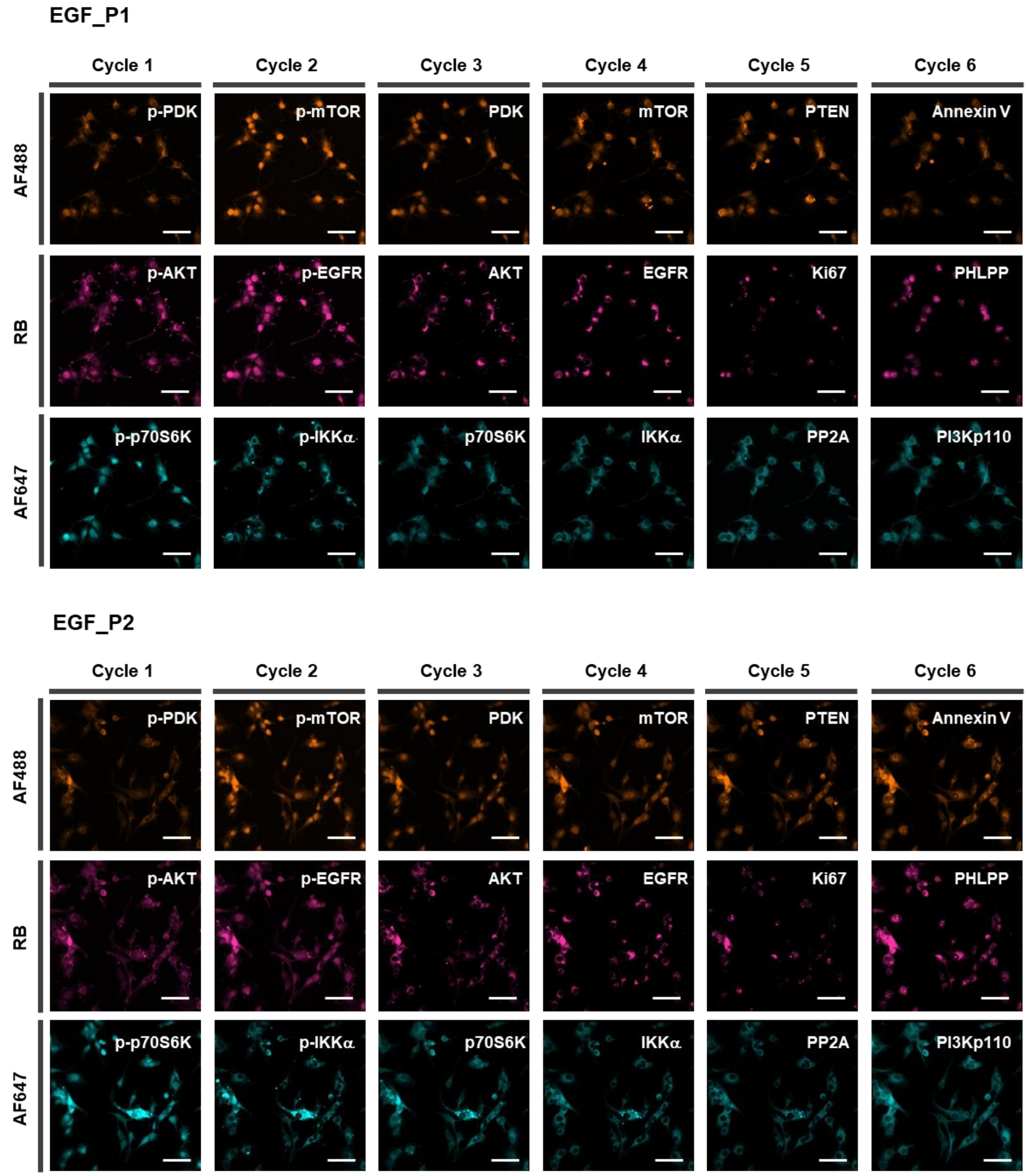

Figure S22. Representative confocal images showing six cycles of PACIFIC on 18 protein targets in EGF-stimulated samples.

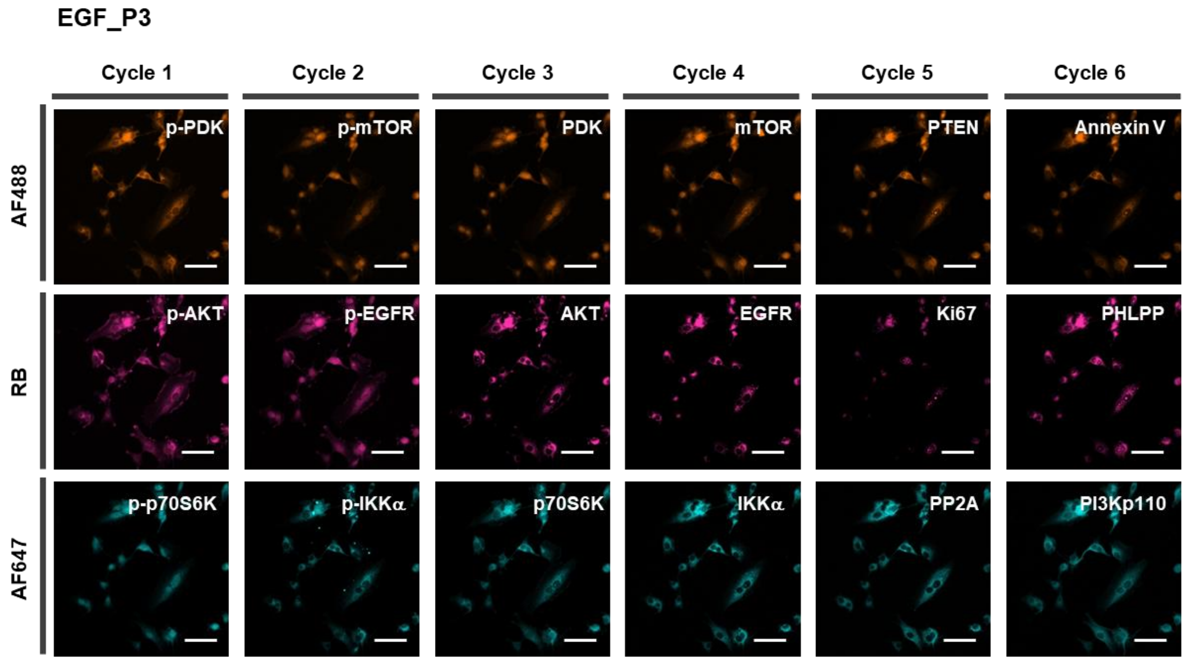

Figure S22 (continued). Representative confocal images showing six cycles of PACIFIC on 18 protein targets in EGF-stimulated samples.

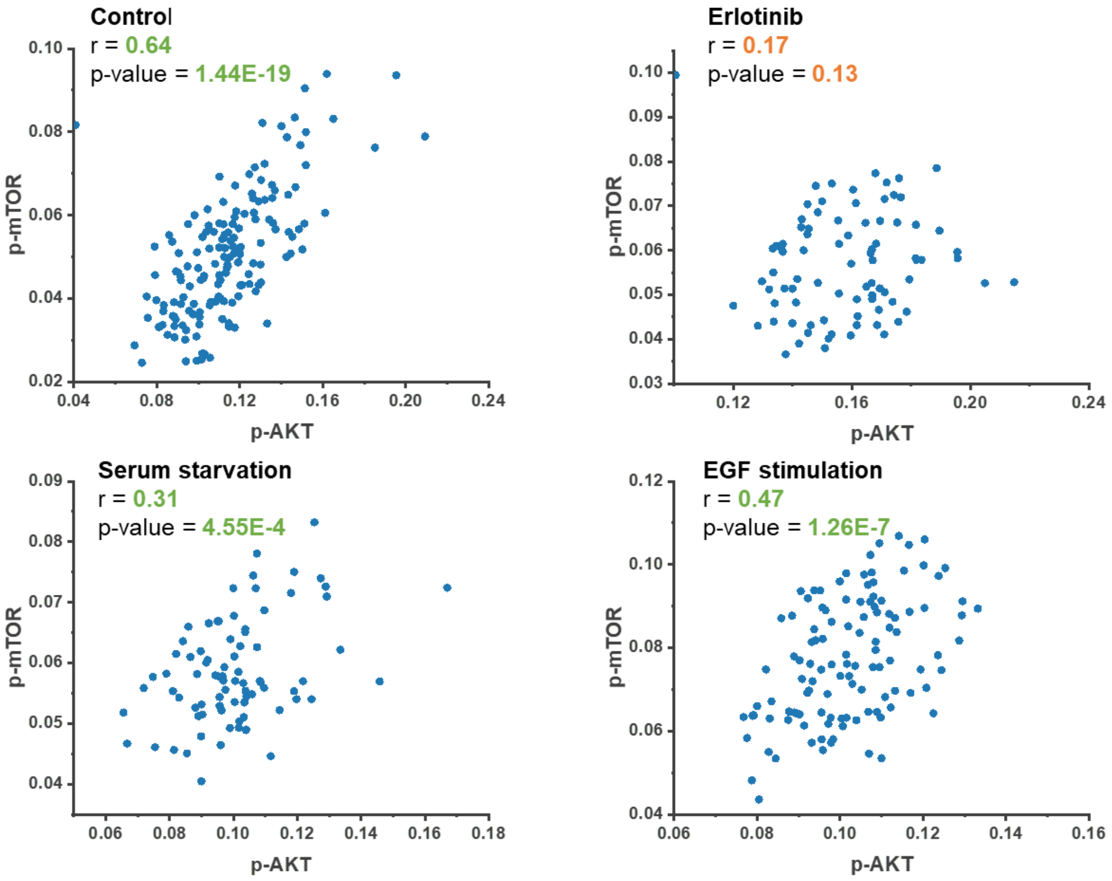

Figure S23. Spearman correlation plots between the p-AKT and p-mTOR under different conditions. The strong correlation between p-AKT and p-mTOR diminished upon EGFR inhibition (Erlotinib) and serum starvation but was rescued by EGF stimulation.

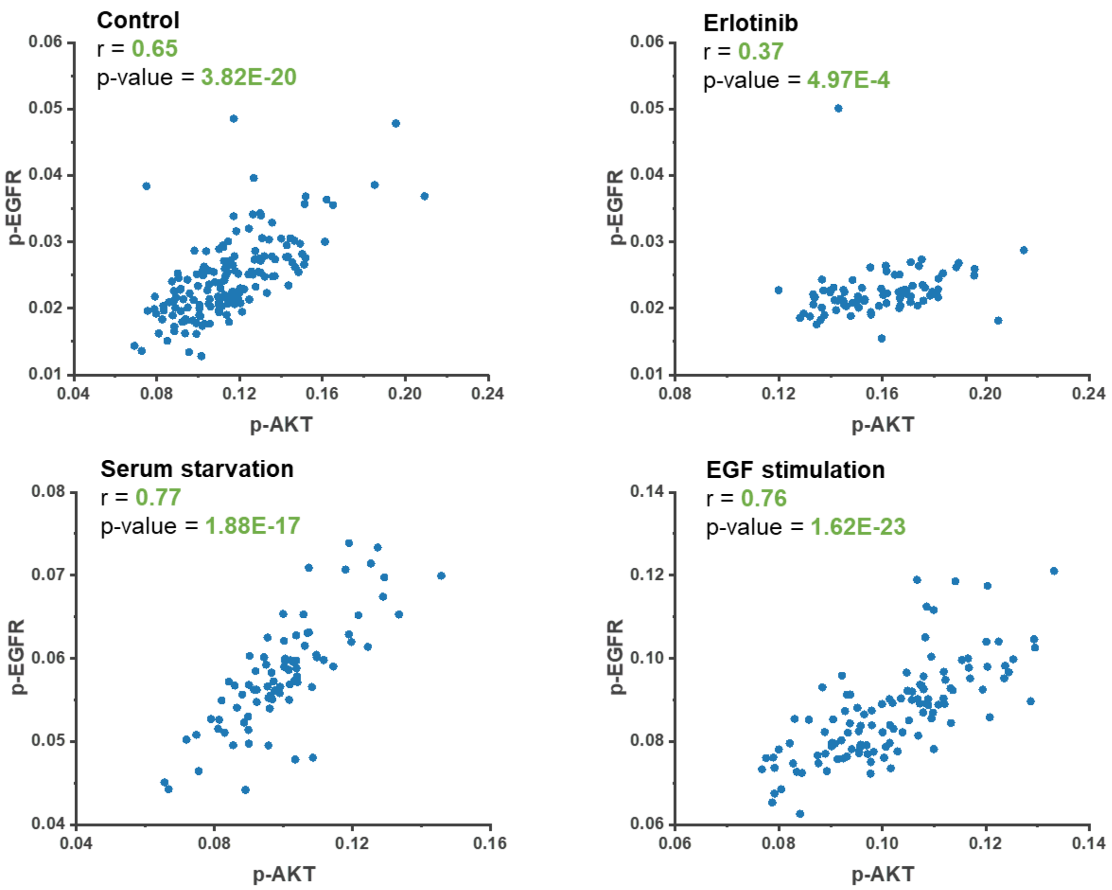

Figure S24. Spearman correlation plots between the p-AKT and p-EGFR under different conditions. The strong correlation between p-AKT and p-EGFR was only inhibited by Erlotinib treatment.

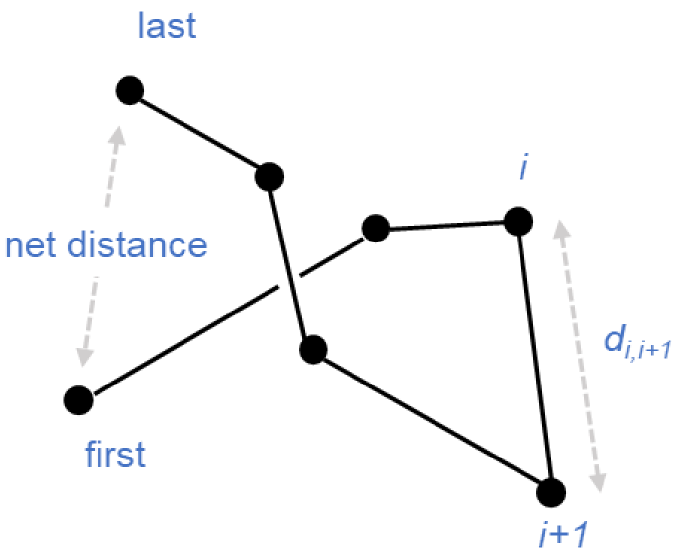

$$total\ distance = \sum d_{i,i+1}$$

$$confinement\ ratio = \frac{net\ distance}{total\ diatance}$$

Figure S25. Confinement ratio. Confinement ration is defined as the net distance divided by total distance traveled. It is a unitless value that ranges from 0 to 1. Values close to 0 indicate a confined movement, where the particle would stay close to its starting point. Values close to 1 indicate that the particle travels along a line with a constant orientation.

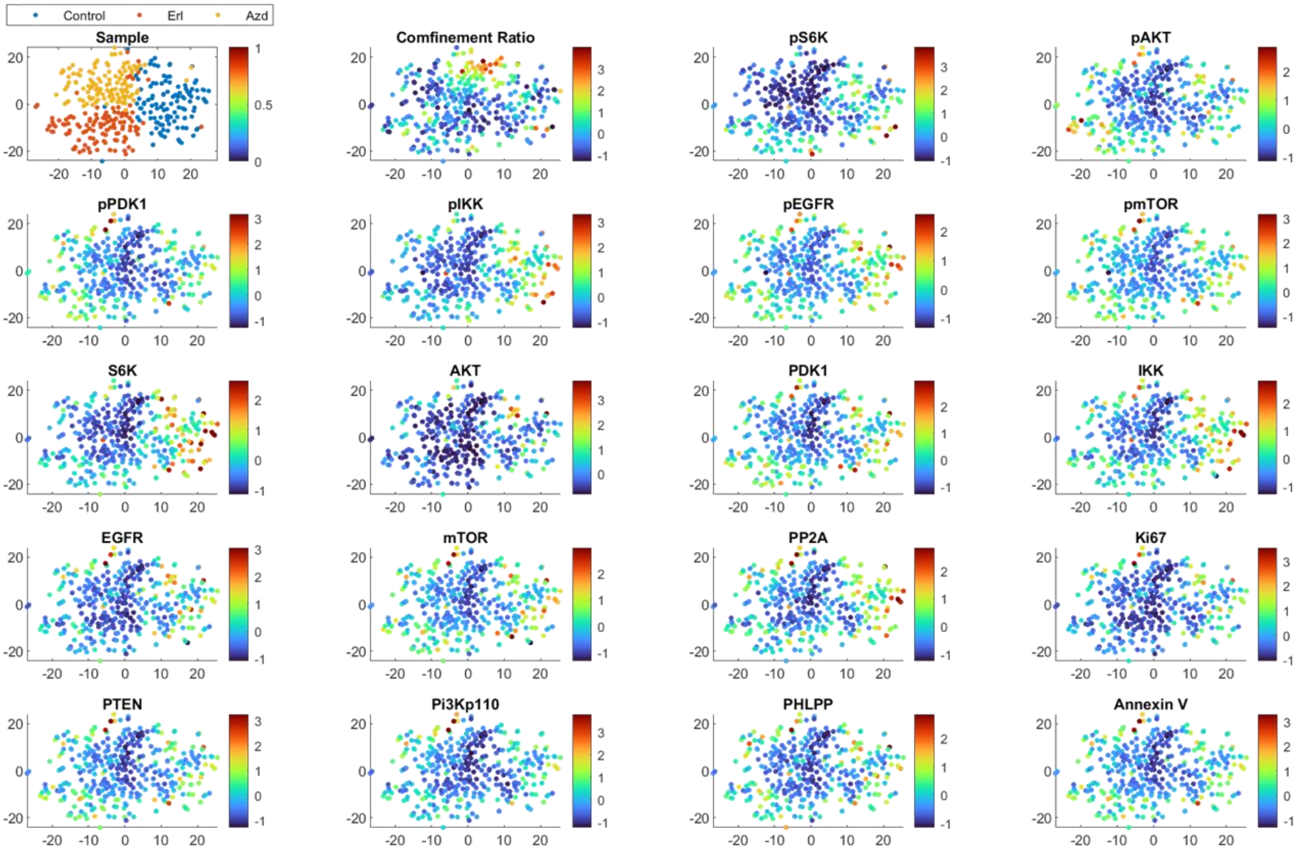

Figure S26. t-SNE plots from single-cell dataset that combining protein expression levels and confinement ratio. Relative analyte intensities are represented as heatmap.

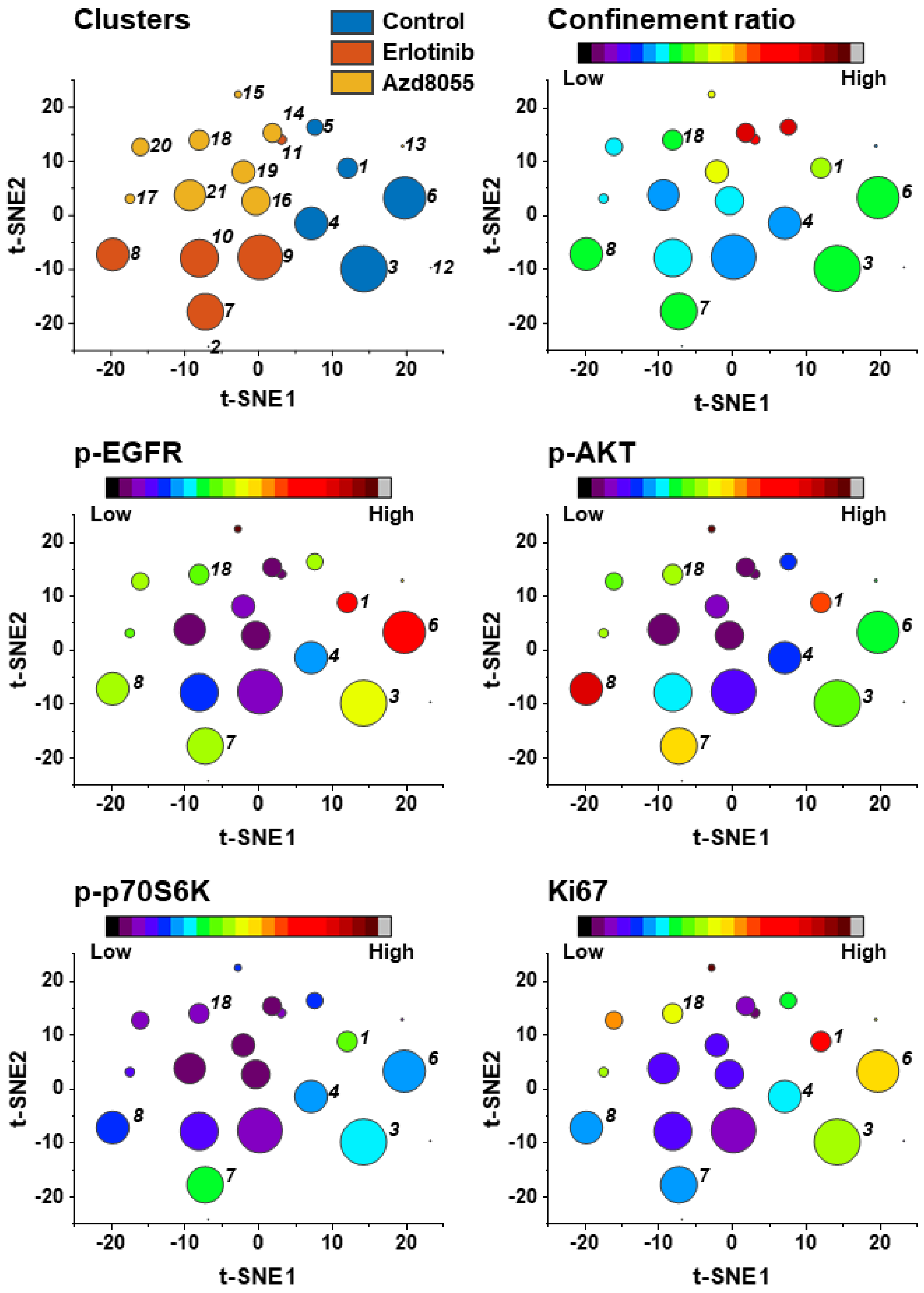

Figure S27. Phenotypical subpopulations derived from the single-cell data by t-SNE analysis followed by the nearest neighbor clustering. The color in the **Clusters** panel indicates different perturbations, and for the rest of the panels, the color indicates the intensity of the analyte.

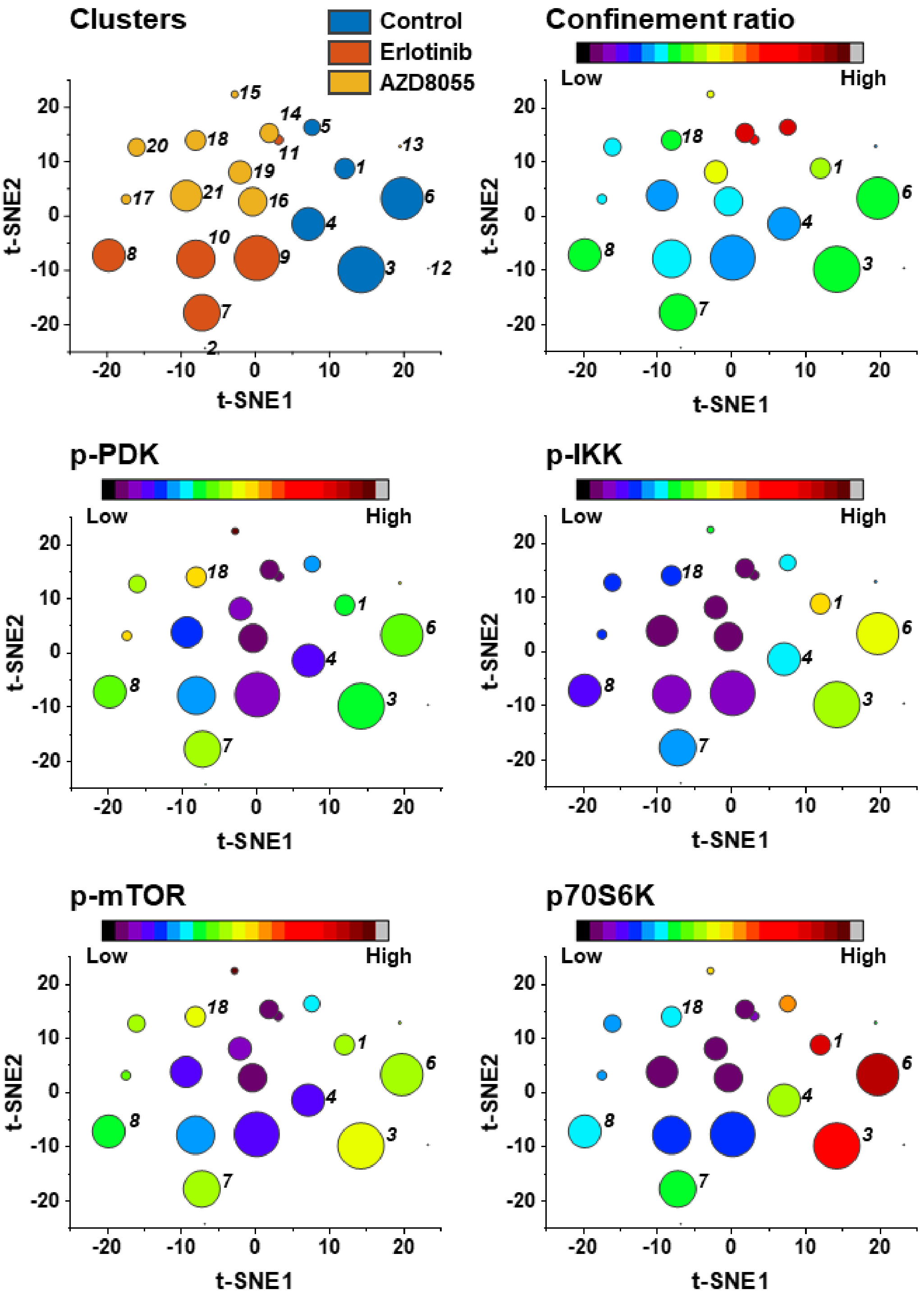

Figure S27 (continued). Phenotypical subpopulations derived from the single-cell data by t-SNE analysis followed by the nearest neighbor clustering. The color in the **Clusters** panel indicates different perturbations, and for the rest of the panels, the color indicates the intensity of the analyte.

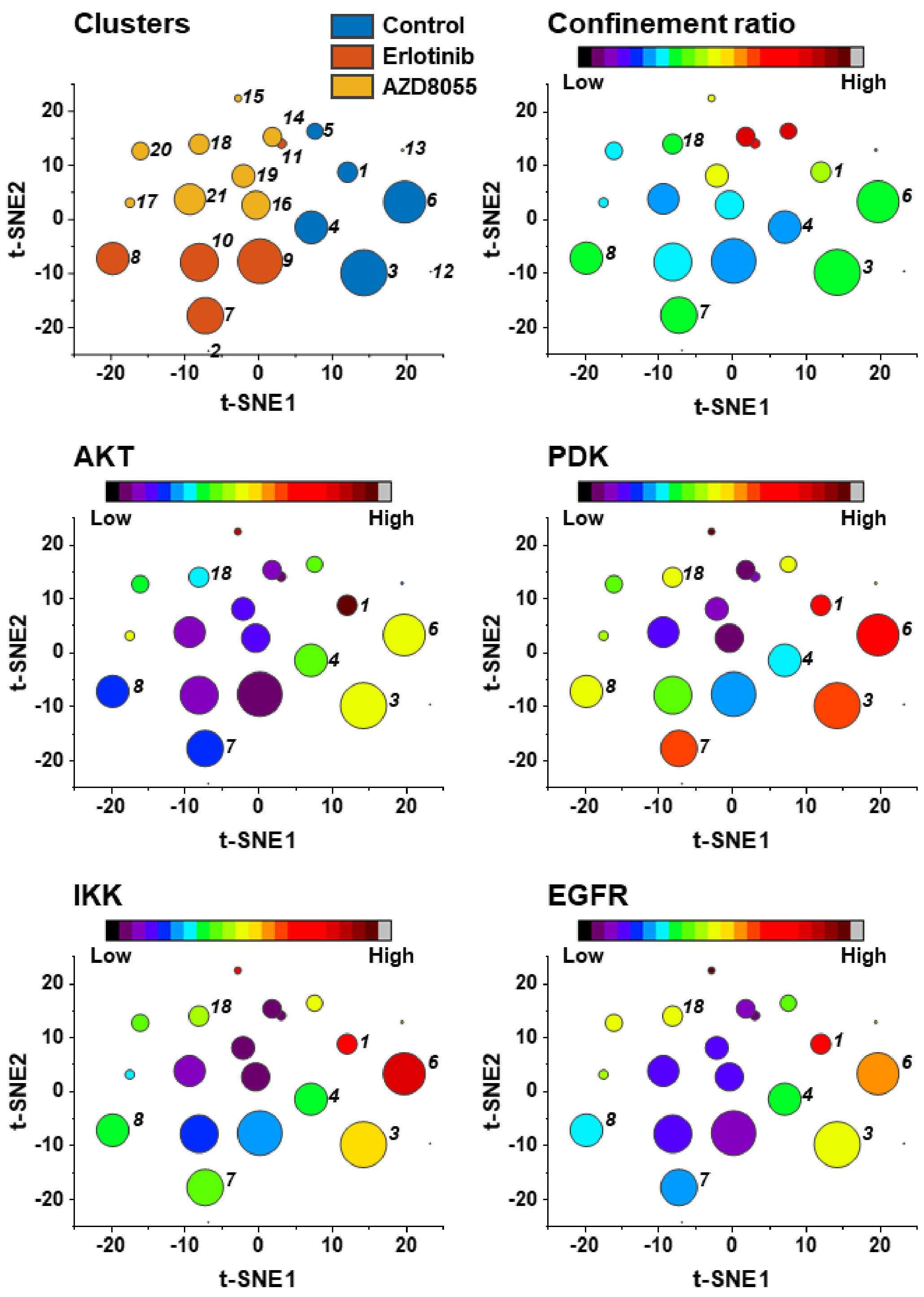

Figure S27 (continued). Phenotypical subpopulations derived from the single-cell data by TSNE analysis followed by the nearest neighbor clustering. The color in the **Clusters** panel indicates different perturbations, and for the rest of the panels, the color indicates the intensity of the analyte.

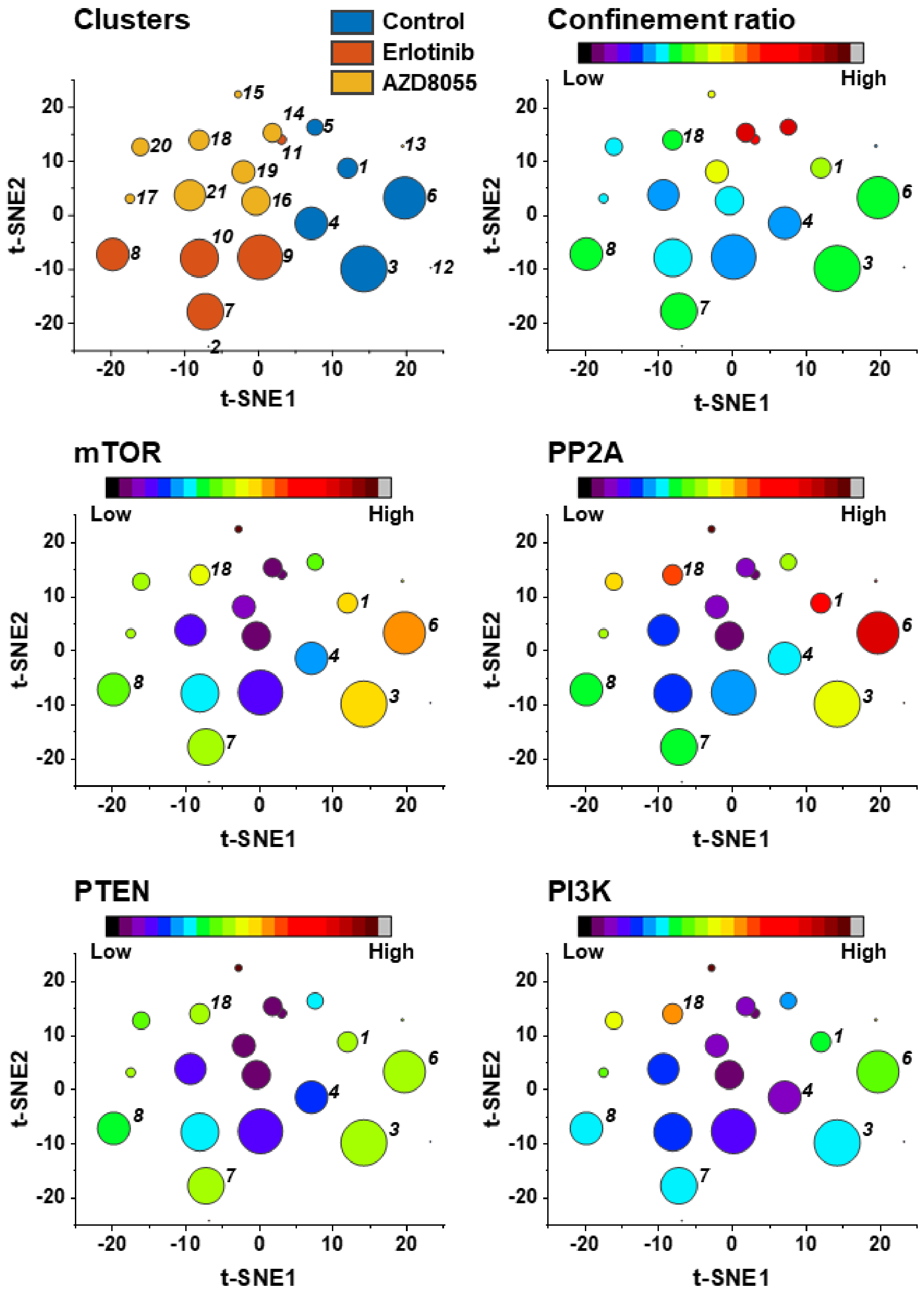

Figure S27 (continued). Phenotypical subpopulations derived from the single-cell data by TSNE analysis followed by the nearest neighbor clustering. The color in the **Clusters** panel indicates different perturbations, and for the rest of the panels, the color indicates the intensity of the analyte.

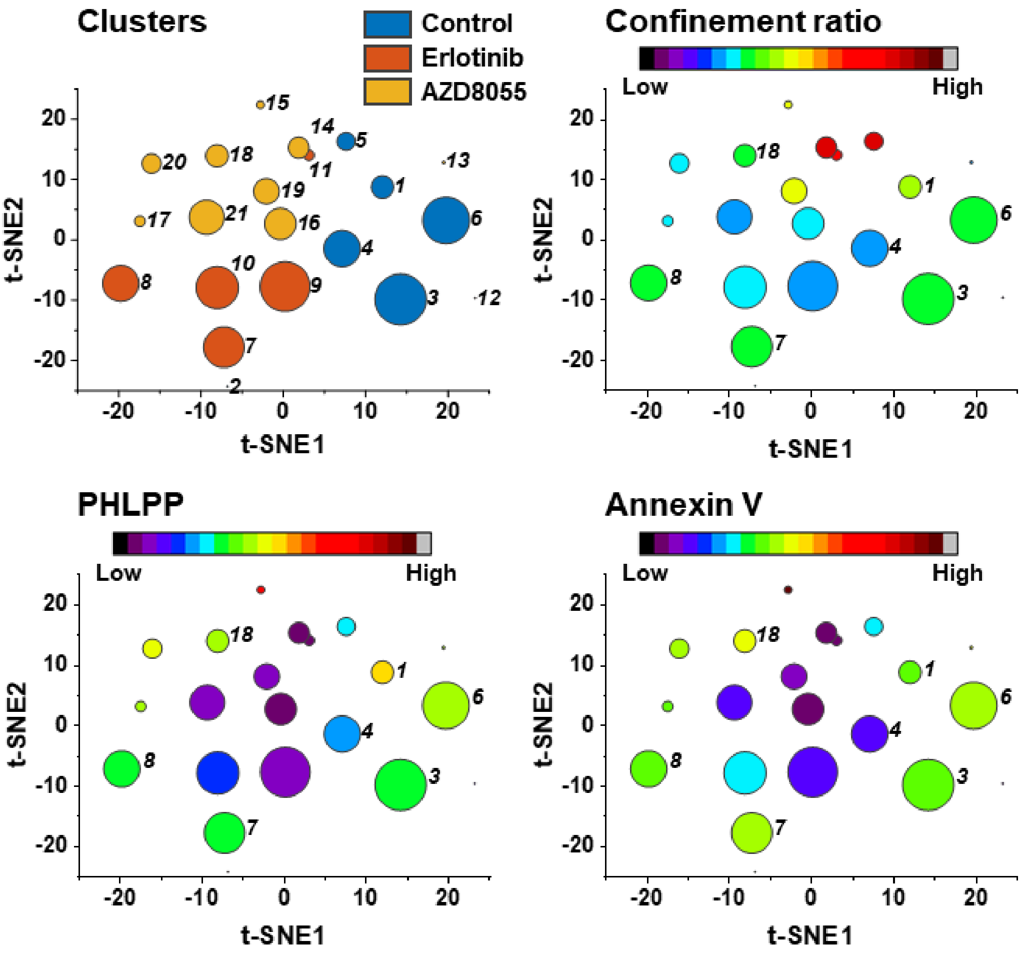

Figure S27 (continued). Phenotypical subpopulations derived from the single-cell data by TSNE analysis followed by the nearest neighbor clustering. The color in the **Clusters** panel indicates different perturbations, and for the rest of the panels, the color indicates the intensity of the analyte.

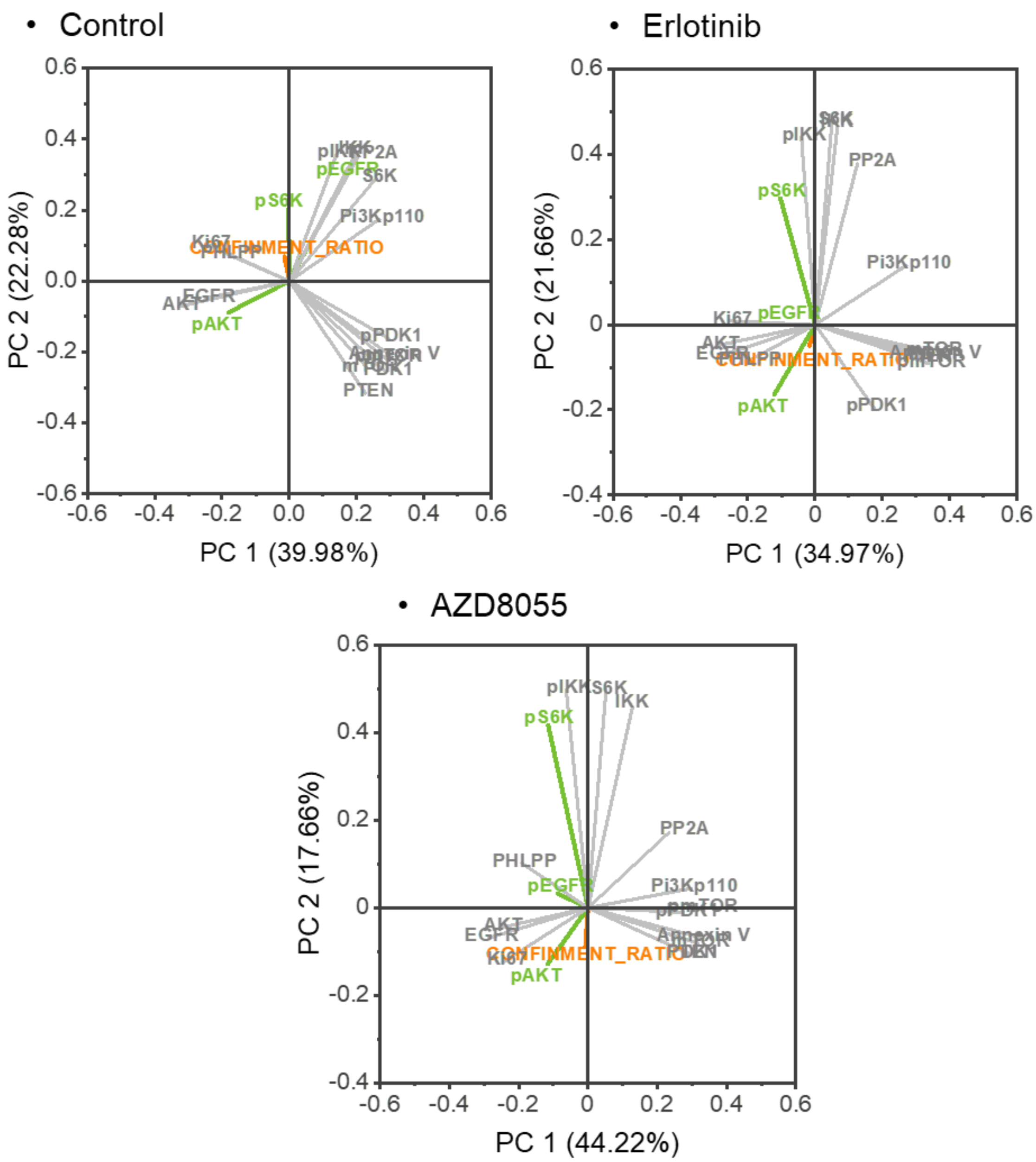

Figure S28. Principle component analysis of the single-cell dataset. In the control sample, confinement ratio exhibited a strong alignment with the p-p70S6K, but it was decoupled in both Erlotinib and AZD8055 conditions.
